# Aromatic Polyketides from the Deep-Sea Cold-Seep Mussel Associated Endozoic Fungus *Talaromyces minioluteus* CS-138

**DOI:** 10.3390/md20080529

**Published:** 2022-08-18

**Authors:** Qi Song, Sui-Qun Yang, Xiao-Ming Li, Xue-Yi Hu, Xin Li, Bin-Gui Wang

**Affiliations:** 1 CAS and Shandong Province Key Laboratory of Experimental Marine Biology, Institute of Oceanology, Chinese Academy of Sciences, Nanhai Road 7, Qingdao 266071, China; 2 Laboratory of Marine Biology and Biotechnology, Qingdao National Laboratory for Marine Science and Technology, Wenhai Road 1, Qingdao 266237, China; 3 College of Marine Science, University of Chinese Academy of Sciences, Yuquan Road 19A, Beijing 100049, China; 4 Center for Ocean Mega-Science, Chinese Academy of Sciences, Nanhai Road 7, Qingdao 266071, China

**Keywords:** *Talaromyces minioluteus*, *Gigantidas platifrons*, deep-sea cold seep, antimicrobial activity, DPPH scavenging activity

## Abstract

Five new aromatic polyketides, including a unique benzofuran derivative, talarominine A (**1**), and four chromone analogs talamins A–D (**2**–**5**), along with one known related metabolite, 5-hydroxy-7-methoxy-2,3-dimethylchromone (**6**), were isolated and identified from the *Talaromyces minioluteus* CS-138, an endozoic fungus obtained from the deep-sea cold seep mussel *Gigantidas platifrons*. Their chemical structures were elucidated by detailed analysis of their NMR spectra, HRESIMS and X-ray crystallographic data, and by comparison with literature data as well. The antibacterial and DPPH scavenging activities of compounds **1**–**6** were evaluated. Compounds **1**–**3** showed inhibitory activity against some of the tested bacteria whereas compounds **2** and **5** showed potent DPPH radical scavenging activities, which were better than that of the positive control butylated hydroxytoluene (BHT). This work is likely the first report on marine natural products of mussel-derived fungus living in cold seep environments.

## 1. Introduction

As most of the marine invertebrates are sessile, soft-bodied, and slow to move, they might need complex secondary metabolites that might be produced by interactions with their symbiotic microorganisms to establish a unique chemical defense system against potential parasite predation and/or harmful microbial colonization. It has been proved that the real producer of a lot of marine natural products isolated from macroorganisms seems to be symbiotic microorganisms, instead of the macrobiota themselves [1]. *Gigantidas platifrons* (deep-sea mussel) belongs to the subfamily Bathymodiolinae (Bivalvia: Mytilidae). It is the most typical macroinvertebrate in the global deep-sea cold seep environments [2]. Deep-sea cold seep is a unique marine environment with a high concentration of methane and low-temperature [3]. In order to survive in this extreme environment, the microorganisms from deep-sea mussel might evolve a more specific metabolic mechanism to produce unique secondary metabolites, which will greatly enrich the research of marine natural products [4].

**Figure 1:**
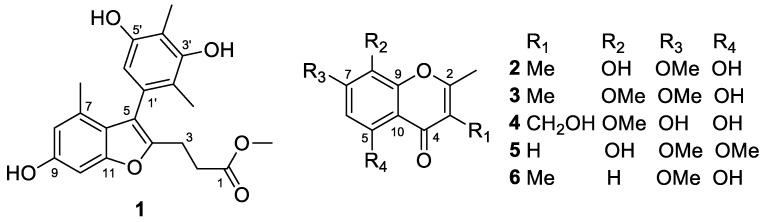
Structures of the isolated compounds **1**–**6** from *T. minioluteus* CS-138.

In our continuing research on secondary metabolites of deep-sea-derived fungi [5,6,7,8], an endozoic fungus *Talaromyces minioluteus* CS-138 was isolated from the inner fresh tissue of *Gigantidas platifrons*, a deep-sea mussel collected from the cold seep area in the South China Sea. The fungal species *T*. *minioluteus* is widely distributed in various environments and has been reported to produce prolific bioactive metabolites, such as antifungal tetracyclic diterpenes, polyketide-terpenoid hybrids, and hydrazide derivatives [9]. In the present work, the HPLC analysis of the EtOAc extract of *T*. *minioluteus* CS-138 showed a series of peaks of typical aromatic polyketides with similar UV absorptions which were not found in our HPLC-UV database. We thus carried out a larger-scale fermentation of *T. minioluteus* CS-138 for chemical investigation. As a result, six secondary metabolites (Figure 1), including a unique benzofuran derivative talarominine A (**1**) and four chromone analogs talamins A–D (**2**–**5**), together with a known related compound, 5-hydroxy-7-methoxy-2,3-dimethylchromone (**6**) [10], were isolated and identified. Chemical structures of compounds **1**–**6** were elucidated by detailed analysis of the spectroscopic data, and the structures of compounds **2**, **4**, and **5** were further confirmed by single-crystal X-ray diffraction analysis. In this article, the isolation, structure identification, antimicrobial activities, and DPPH scavenging activities of compounds **1**–**6** were elaborated. The work described in the manuscript appears to be the first report on marine natural products from deep-sea cold seep *Gigantidas platifrons*-derived fungus.

## 2. Results and Discussion

### 2.1. Structure Elucidation of the New Compounds

Compound **1** was obtained as a yellow amorphous solid, and its molecular formula was determined as C_21_H_22_O_6_ by analyzing the HRESIMS data at *m/z* 369.1341 [M − H]^−^ (calculated for C_21_H_21_O_6_, 369.1344, Figure S1 in the Supplementary Materials), indicating 11 degrees of unsaturation. The ^1^H NMR data of **1** (DMSO-*d*_6_, Table 1 and Figure S2) displayed signals for four methyls (including one methoxyl), three aromatic protons, and three exchangeable protons. The ^13^C NMR and DEPT data (DMSO-*d*_6_, Table 1 and Figure S3) revealed the presence of 21 carbons, including four methyls (with one oxygenated), two sp^3^-hybridized methylenes, three sp^2^-hybridized methines, and 12 non-protonated carbons (including five *O*-bearing carbons and one ester carbonyl carbon). 

**Figure 2:**
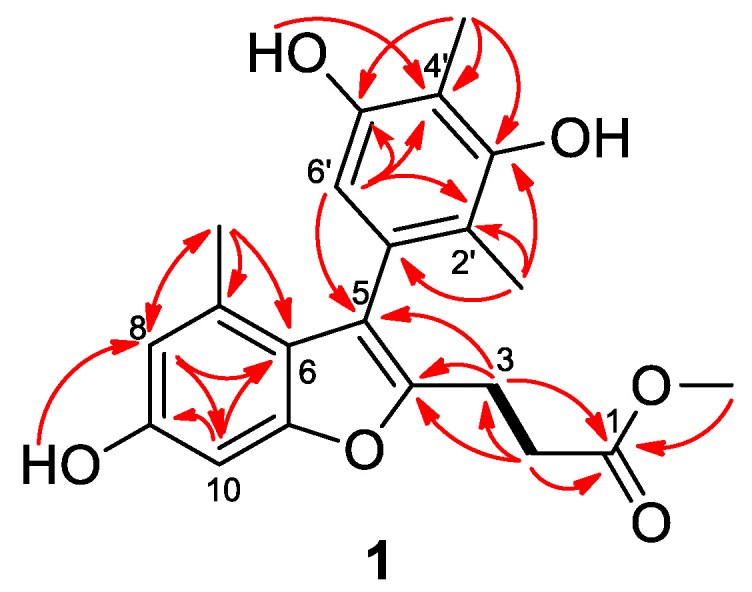
Key COSY (bold lines) and HMBC (red arrows) correlations for compound **1**.

The structure of compound **1** was further identified by detailed analysis of ^1^H-^1^H COSY and HMBC data (Figure 2). HMBC correlations from H-8 to C-6 and C-10 and from H-10 to C-6, C-8, and C-9 indicated a 1,2,3,5-tetrasubstituted benzene ring in compound **1**. Furthermore, a methyl group at C-7 of the benzene ring was confirmed by the HMBC correlations from CH_3_-7 (*δ*_H_ 1.86) to C-6, C-7, and C-8, whereas a hydroxy group at C-9 was established by the HMBC correlations from the proton of OH-9 to C-8 (Figure 2). In addition, a pentasubstituted benzene ring in **1** was confirmed by the HMBC correlations from H-6′ to C-2′, C-4′, and C-5′. Two methyl groups were attached to C-2′ and C-4′ of the pentasubstituted benzene ring as evidenced by the HMBC correlations from CH_3_-2′ (*δ*_H_ 1.79) to C-1′, C-2′, and C-3′ and from CH_3_-4′ (*δ*_H_ 2.04) to C-3′, C-4′, and C-5′ (Figure 2). Supported by the HMBC correlations from OH-5′ to C-4′ and the chemical shift of *δ*_C_ 153.7 (C-3′), the locations of two hydroxy groups were designated at C-5′ and C-3′, respectively. The side chain was determined by the HMBC cross peaks from the proton of a methoxy group at *δ*_H_ 3.55 (3H, s) to C-1, from H_2_-3 and H_2_-2 to C-1, and by the COSY correlations from H_2_-3 to H_2_-2 (Figure 2). 

The chemical shifts of *δ*_C_ 154.7 (C-11), 149.8 (C-4), 116.9 (C-5), and 119.5 (C-6) and the HMBC cross peaks from H-2 to C-4 and from H-6′ and H-3 to C-5 determined that the 1,2,3,5-tetrasubstituted benzene ring and the side chain were connected to a furan ring through C-5 and C-4, respectively (Figure 2). Thus, the structure of compound **1** was determined as a unique benzofuran derivative and was given the trivial name talarominine A. A literature survey revealed that talarominine A is a new naturally occurring benzofuran derivative with unique substitution patterns.

**Figure 3:**
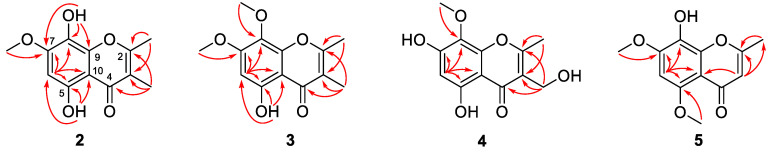
Key HMBC correlations (arrows) for compounds **2**–**5**.

**Figure 4:**
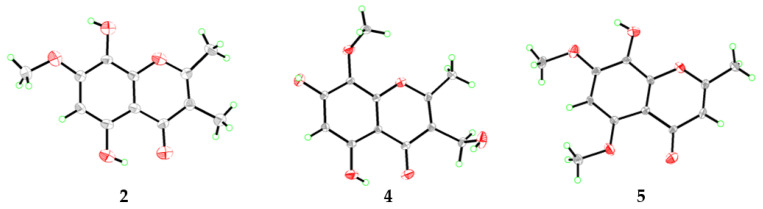
X-ray crystal structures of compounds **2**, **4**, and **5**.

Compound **2** was obtained as yellow crystals and its molecular formula was determined to be C_12_H_12_O_5_ on the basis of HRESIMS data at *m/z* 237.0762 [M + H]^+^ (calculated for C_12_H_13_O_5_, 237.0757, Figure S7), requiring 7 degrees of unsaturation. The ^1^H (DMSO-*d*_6_, Table 2 and Figure S8) and ^13^C (DMSO-*d*_6_, Table 3 and Figure S9) NMR data revealed the presence of three methyls (one oxygenated), one methine, and eight non-protonated carbons, which were quite similar to those of previously reported 5,7-dihydroxy-6-methoxy-2-methylchromone [11]. Discreet analysis and comparison of the NMR data disclosed that compound **2** and 5,7-dihydroxy-6-methoxy-2-methylchromone shared the same benzopyran-4-one ring but with different substituents. HMBC correlations from the proton at *δ*_H_ 6.49 (1H, s, H-6) to C-5, C-7, C-8, and C-10 led to the designation of this proton as H-6 (Figure 3), whereas the methyl groups were assigned at C-2 and C-3 by the HMBC cross peaks from CH_3_-2 to C-2 and C-3 and from CH_3_-3 to C-2, C-3, and C-4. Moreover, HMBC correlations from OCH_3_-7 to C-7, from OH-8 to C-7, C-8, and C-9, and from OH-5 to C-5, C-6, and C-10 designated the locations of the methoxy and two hydroxy groups (Figure 3). Furthermore, the structure of compound **2** was confirmed by single-crystal X-ray diffraction experiment (Figure 4). Thus, compound **2** was determined as 5,8-dihydroxy-7-methoxy-2,3-dimethylchromone and was named as talamin A.

Compound **3** was also obtained as yellow powder, and its molecular formula was determined to be C_13_H_14_O_5_ by HRESIMS data at *m*/*z* 251.0912 [M + H]^+^ (calculated for C_13_H_15_O_5_, 251.0914, Figure S13), with 14 units more than that of **2** but having same degrees of unsaturation with **2**. The ^1^H NMR data (DMSO-*d*_6_, Table 2 and Figure S14) was similar to that of compound **2**, except that the hydroxy group at C-8 of **2** was replaced by a methoxy group in **3**, and the ^13^C NMR data (DMSO-*d*_6_, Table 3 and Figure S15) and HMBC correlations (Figure 3) confirmed this designation. Thus, compound **3** was determined as 5-hydroxy-7,8-dimethoxy-2,3-dimethylchromone and gave the trivial name talamin B.

Compound **4** was also isolated as yellow crystals. Its molecular formula, C_12_H_12_O_6_, was determined by HRESIMS at *m*/*z* 275.0520 [M + Na]^+^ (calculated for C_12_H_12_O_6_Na, 275.0526, Figure S18). Compared to **2**, compound **4** has one less methyl signal and one more oxygenated methylene signal from the ^1^H and ^13^C NMR data (DMSO-*d*_6_, Table 2 and Table 3 and Figures S19 and S20). HMBC correlations (Figure 3) from the methylene proton of 3-CH_2_OH (*δ*_H_ 4.35) to C-2, C-3, and C-4 designated the location of the oxygenated methylene group at C-3. In addition, the chemical shifts of the carbon atoms of **4** at benzene ring have changed greatly when compared with that of compound **2**, which was speculated to be caused by the different substitution patterns of the methoxy and hydroxy groups on the benzene ring. HMBC correlations from the proton of OCH_3_-8 (*δ*_H_ 3.74) to C-8 and the chemical shifts of *δ*_C_ 159.7 (C-7) verified the locations of the hydroxy and methoxyl groups. After the NMR data collection, we set **4** to single-crystal X-ray diffraction experiment and confirmed our deduction (Figure 4). Thus, compound **4** was assigned as a new chromone derivative and was named as talamin C.

Compound **5** was obtained as light yellow crystals and its molecular formula was assigned as C_12_H_12_O_5_ by HRESIMS at *m*/*z* 235.0614 [M − H]^−^ (calculated for C_12_H_11_O_5_, 235.0612, Figure S24), the same as that of compound **2**. The ^1^H NMR data (DMSO-*d*_6_, Table 2 and Figure S25) of **5** was similar to that of **2**. However, the olefinic-methyl group at C-3 and the OH group at C-5 in **2** were replaced by an olefinic proton and a methoxy group in **5**, respectively. HMBC correlations (Figure 3) from *δ*_H_ 5.90 (1H, s, H-3) to C-2 and C-10 designated this proton as H-3. In addition, chemical shifts of the carbonyl and several aromatic carbon atoms changed significantly, which indicated that the substituent on benzene ring has changed. HMBC correlations from OCH_3_-7 (*δ*_H_ 3.92) to C-7 and from OCH_3_-5 (*δ*_H_ 3.79) to C-5 verified the locations of the methoxy groups. The single-crystal X-ray diffraction experiment (Figure 4) further verified the locations of the hydroxy and methoxy groups. Thus, compound **5** was identified as 8-hydroxy-5,7-dimethoxy-2-methylchromone and was named as talamin D.

### 2.2. Antibacterial Assays

All of the isolated compounds were tested for antibacterial activities against three humanic and 9 aquatic pathogenic bacteria. As shown in Table 4, compound **1** exhibited inhibitory activities against methicillin-resistant *Staphylococcus aureus* (MRSA), *Micrococcus luteus*, *Pseudomonas aeruginosa*, *Vibrio harveyi*, and *Vibrio vulnificus*, with MIC values ranging from 32 to 64 μg/mL. Compound **2** showed certain activity against *V. vulnificus* with an MIC value of 32 μg/mL, whereas compound **3** exhibited inhibitory activities against MRSA and *V. vulnificus*. For both, the MIC values were 64 μg/mL. Compounds **4**–**6** showed no or weak activity to the tested strains (MIC > 64 μg/mL). 

### 2.3. DPPH Scavenging Activities

Compounds **1**–**6** were further evaluated for their 2,2-diphenyl-1-picrylhydrazyl (DPPH) radical scavenging activity. The results (Table 5) showed that compounds **1**, **2**, and **5** had strong DPPH radical scavenging activities, with IC_50_ values 24.10, 6.56, and 9.03 μM, which are significantly better than that of the positive control BHT (IC_50_ = 61.39 μM). A structure activity relationship analysis of compounds **2**–**6** indicated that the hydroxy group at C-8 of compounds **2** and **5** was essential for their antioxidant property.

## 3. Experimental Section

### 3.1. General Experimental Procedures

One-dimensional and 2D NMR date was acquired on a Bruker Avance 500 spectrometer (Bruker Biospin Group, Karlsruhe, Germany). UV spectra were read from a PuXi TU-1810 UV-visible spectrophotometer (Shanghai Lengguang Technology Co., Ltd., Shanghai, China). Mass spectra were recorded on an API QSTAR Pulsar 1 mass spectrometer (Applied Biosystems, Foster City, CA, USA). Analytical HPLC was performed using a SHIMADZU prominence HPLC system equipped with LC-20AT pump, SIL-20A automated sample injector, CTO-20AC colomn oven, and SPD-M20A diode array detector controlled by LCSolution software.

Column chromatography (CC) was used with silica gel (200–300 mesh, Qingdao Haiyang Chemical Factory, Qingdao, China), Lobar LiChroprep RP-18 (40–60 μm, Merck, Darmstadt, Germany), and Sephadex LH-20 (18–110 μm, Merck, Germany). Thin Layer Chromatography (TLC) was performed with silica gel GF254 precoated plates (50 × 100 mm, Qingdao Haiyang Chemical Group Corporation, Qingdao, China). A separation and purification experiment was carried out with distilled organic solvents.

### 3.2. Fungal Material

The fungal strain *Talaromyces minioluteus* CS-138 was isolated from the inner fresh tissue of the *Gigantidas platifrons*, which is a deep-sea mussel collected from the cold seep area of the south Sea of China in July 2018. After strain identification with the morphological character and ITS region sequence [12], it was found that the fungal strain was the same (100%) as that of *Talaromyces minioluteus* (JX091487). The strain (sequence in GenBank with acccession No. OM670209) is preserved at the Key Laboratory of Experimental Marine Biology, Institute of Oceanology, Chinese Academy of Sciences (IOCAS).

### 3.3. Fermentation, Extraction, and Isolation

For chemical investigations, fresh mycelia of *T. minioluteus* CS-138 were grown on PDA medium with seawater at 28 °C for five days. Then, the strain was inoculated on rice medium for large-scale fermentation in 130 × 1 L Erlenmeyer flasks (70 g rice, 0.1 g corn syrup, 0.3 g peptone, 0.1 g methionine, and 100 mL naturally sourced and filtered seawater) and statically cultured for 30 days at room temperature.

The fermentation product was first extracted with ethyl acetate three times, then extracted with acetone solvent one time, and finally about 120 g of crude extract was obtained. The crude extract was simply segmented by vacuum liquid chromatography (VLC) with different solvents upon the polarity, obtaining nine fractions (Frs. 1–9). Fr. 4 (12.6 g) and Fr. 6 (13.3 g), which showed a series of polyketones as analyed by HPLC and TLC, were further segmented by column chromatography (CC) over Lobar LiChroprep RP-18 with a MeOH–H_2_O gradient (from 10: 90 to 100: 0) to afford subfractions (Fr.4.1–4.10 and Fr.6.1–6.5, respectively). Fr. 6.4 (134.8 mg) was further purified by preparative TLC (pTLC) (plate: 20 × 20 cm, developing solvents: CH_2_Cl_2_/MeOH, 20:1) and then by CC on Sephadex LH-20 (MeOH) to obtain compound **1** (4.1 mg). Fr. 4.3 was further purified by CC on Si gel eluting with a CH_2_Cl_2_-MeOH gradient (from 200:1 to 20:1) and on Sephadex LH-20 (MeOH) to obtain compounds **2** (12.1 mg), **3** (7.8 mg), and **4** (5.3 mg). Similarly, Fr. 6.3 (89.3 mg) was further purified by CC on Si gel eluting with a CH_2_Cl_2_-MeOH gradient (from 170:1 to 20:1) and on Sephadex LH-20 (MeOH) to obtain compounds **5** (7.3 mg) and **6** (5.4 mg). 

Talarominine A (**1**): yellow, amorphous powder; UV (MeOH) *λ*_max_ (log *ε*) 205 (4.69), 253 (4.12), 287 (3.89); ^1^H and ^13^C NMR data, see Table 1; HRESIMS at *m/z* 369.1341 [M - H]^−^ (calcd for C_21_H_21_O_6_, 369.1344).

Talamin A (**2**): yellow crystals; mp 207–209 °C; UV (MeOH) *λ*_max_ (log *ε*) 261 (4.36), 348 (3.59); ^1^H and ^13^C NMR data, see Table 2 and Table 3; HRESIMS at *m/z* 237.0762 [M + H]^+^ (calcd for C_12_H_13_O_5_, 237.0757).

Talamin B (**3**): yellow powder; UV (MeOH) *λ*_max_ (log *ε*) 252 (4.22), 332 (3.55); ^1^H and ^13^C NMR data, see Table 2 and Table 3; HRESIMS at *m/z* 251.0912 [M + H]^+^ (calcd for C_13_H_15_O_5_, 251.0914).

Talamin C (**4**): yellow crystals; mp > 350 °C; UV (MeOH) *λ*_max_ (log *ε*) 228 (4.44), 260 (4.60), 326 (3.88); ^1^H and ^13^C NMR data, see Table 2 and Table 3; HRESIMS at *m/z* 275.0520 [M + Na]^+^ (calcd for C_12_H_12_O_6_Na, 275.0526).

Talamin D (**5**): light yellow crystals; mp 227–229 °C; UV (MeOH) *λ*_max_ (log *ε*) 225 (3.37), 260 (3.55), 333 (2.86); ^1^H and ^13^C NMR data, see Table 2 and Table 3; HRESIMS at *m/z* 235.0614 [M − H]^−^ (calcd for C_12_H_11_O_5_, 235.0612).

### 3.4. X-ray Crystallographic Analysis of Compounds ***2***, ***4***, and ***5*** [13]

The crystallographic data of compound **4** were collected on a Bruker Smart-1000 diffractometer equipped with a graphite-monochromatic Cu K*α* radiation (*λ* = 1.54178) Å at 296 (2) K, whereas those of compounds **2** and **5** were collected with a graphite-monochromatic Mo K*α* radiation (*λ* = 0.71073) Å at 293 (2) K and 298 (2) K, respectively. The data were corrected for absorption by using the program SADABS [14]. The structures were solved by direct methods with the SHELXTL software package [15]. All non-hydrogen atoms were refined anisotropically. The H atoms connected to C atoms were calculated theoretically and those to O atoms were assigned by difference Fourier maps. The structures were optimized by full-matrix least-squares techniques [16].

*Crystal data for compound **2**:* C_12_H_12_O_5_, F.W. = 236.07, Triclinic, space group P-1, unit cell dimensions *a* = 4.6047(4) Å, *b* = 8.4916(8) Å, *c* = 13.8731(11) Å, *V* = 539.12(8) Å^3^, *α* = 89.525(2)°, *β* = 86.7350(10)°, *γ* = 84.5400(10)°, *Z* = 2, *d*_calcd_ = 1.444 mg/m^3^, crystal dimensions 0.30 × 0.13 × 0.05 mm, *μ* = 0.045 mm^–1^, *F*(000) = 72. The 2748 measurements yielded 1857 independent reflections after equivalent data were averaged. The final refinement gave *R*_1_ = 0.0872 and w*R*_2_ = 0.2001 [*I* > 2*σ*(*I*)].

*Crystal data for compound **4**:* C_12_H_12_O_6_, F.W. = 252.22, Triclinic, space group P-1, unit cell dimensions *a* = 7.0563(10) Å, *b* = 9.0035(13) Å, *c* = 11.8983(17) Å, *V* = 722.36(18) Å^3^, *α* = 81.350(4)°, *β* = 75.651(4)°, *γ* = 84.160(4)°, *Z* = 2, *d*_calcd_ = 1.160 mg/m^3^, crystal dimensions 0.180 × 0.160 × 0.150 mm, *μ* = 0.806 mm^–1^, *F*(000) = 264. The 12,637 measurements yielded 2616 independent reflections after equivalent data were averaged. The final refinement gave *R*_1_ = 0.0872 and w*R*_2_ = 0.2001 [*I* > 2*σ*(*I*)].

*Crystal data for compound **5**:* C_12_H_12_O_5_, F.W. = 236.22, monoclinic, space group C2/c, unit cell dimensions *a* = 21.326(2) Å, *b* = 6.1455(8) Å, *c* = 16.7254(19) Å, *V* = 2182.7(5) Å^3^, *α* = *γ* = 90.00°, *β* = 95.304(2)°, *Z* = 8, *d*_calcd_ = 1.438 mg/m^3^, crystal dimensions 0.35 × 0.17 × 0.10 mm, *μ* = 0.113 mm^–1^, *F*(000) = 992. The 4926 measurements yielded 1896 independent reflections after equivalent data were averaged. The final refinement gave *R*_1_ = 0.0934 and w*R*_2_ = 0.2083 [*I* > 2*σ*(*I*)].

### 3.5. Antibacterial Assay

Antibacterial activities against three human pathogens (*Escherichia coli* EMBLC-1*,* methicillin-resistant *Staphylococcus aureus* EMBLC-2, and *Micrococcus luteus* QDIO-3) and 9 aquatic bacteria (*Aeromonas hydrophila* QDIO-1, *Klebsiella pneumonia* EMBLC-3, *Edwardsiella* *tarda* QDIO-2, *Pseudomonas aeruginosa* QDIO-4, *Vibrio alginolyticus* QDIO-5, *V. anguillarum* QDIO-6*, V. harveyi* QDIO-7, *V. parahemolyticus* QDIO-8, and *V. vulnificus* QDIO-10) were carried out by the microplate assay with a microplate assay with three repetitions [17]. The humanic and aquatic pathogens were obtained from the Institute of Oceanology, Chinese Academy of Sciences. Chloramphenicol was used as positive control.

### 3.6. DPPH Radical Scavenging Assay

The scavenging activity against DPPH radicals was carried out according to the method of Sharma with some modifications [18,19]. Certain amounts of compounds **1**–**6** were individually dissolved in methanol and diluted into six gradients. Then, 100 mL aliquot of samples was added to 100 mL of 0.16 mM DPPH methanolic solution in 96-well plates. After mixing evenly, the mixtures were left to stand at room temperature for 30 min in the dark, and the absorbance was read at 517 nm (A_sample_). Butylated hydroxytoluene (BHT) was used as positive control. All the measurements were performed in triplicate and each value was presented as the mean ± standard deviation. The ability to scavenge the DPPH was calculated according to the equation:

Scavenging effect (%) = 100 − (A_sample_ − A_sample blank_) × 100/(A_control_ − A_blank_)

A_sample blank:_: the absorbance of the test sample without DPPH solution;

A_control_: the absorbance of the DPPH solution;

A_blank_: the absorbance of methanol.

## 4. Conclusions

In summary, the secondary metabolites of *T. minioluteus* CS-138, which was isolated from the fresh tissues of *Gigantidas platifrons* in the cold seep area of South China Sea, were chemically studied. Six aromatic polyketides, including five new compounds (**1–5**), were isolated and identified. Among them, compounds **2**, **4**, and **5** were further confirmed by single-crystal X-ray diffraction analysis. Compounds **1**, **2**, and **3** exhibited inhibitory activities against some human pathogenic and aquatic bacteria, with MIC values ranging from 32 to 64 μg/mL. Moreover, compounds **1**, **2**, and **5** exhibited potent DPPH radical scavenging activities, significantly better than that of the positive control BHT, possessing the potential to be developed as antioxidants.

## Figures and Tables

**Table 1:** NMR data of compound **1** (*δ* in ppm, 500 MHz for ^1^H and 125 MHz for ^13^C).

Compound 1 (DMSO-*d*_6_)					
No.	*δ*_H_ (mult, *J* in Hz)	*δ*_C_, Type	No.	*δ*_H_ (mult, *J* in Hz)	*δ*_C_, Type
1		172.1, C	2′		115.0, C
2	2.55, dd (7.9, 6.7)	31.9, CH_2_	3′		153.7, C
3	2.72, m	21.7, CH_2_	4′		110.7, C
4		149.8, C	5′		153.1, C
5		116.9, C	6′	6.21, s	108.8, CH
6		119.5, C	7-CH_3_	1.86, s	17.4, CH_3_
7		130.9, C	2′-CH_3_	1.79, s	13.2, CH_3_
8	6.41, d (2.0)	112.9, CH	4′-CH_3_	2.04, s	9.3, CH_3_
9		154.4, C	9-OH	8.10, s	
10	6.68, d (2.0)	95.1, CH	3′-OH	9.31, s	
11		154.7, C	5′-OH	8.96, s	
1′		130.2, C	1-OCH_3_	3.55, s	51.4, CH_3_

**Table 2:** ^1^H NMR data of compounds **2**–**5** in DMSO-*d*_6_ (*δ* in ppm, 500 MHz for ^1^H).

*δ*_H_ (mult, *J* in Hz)				
No.	2	3	4	5
3				5.90, s
6	6.49, s	6.53, s	6.18, s	6.62, s
2-CH_3_	2.40, s	2.42, s	2.47, s	2.27, s
3-CH_3_/CH_2_	1.90, s	1.91, s	4.35, s	
5-OH	12.48, s	12.82, s	12.65, s	
8-OH	8.71, s			
5-OCH_3_				3.79, s
7-OCH_3_	3.87, s	3.88, s		3.92, s
8-OCH_3_		3.74, s	3.74, s	

**Table 3:** ^13^C NMR data of compounds **2**–**5** in DMSO-*d*_6_ (*δ* in ppm, 125 MHz for ^13^C).

*δ*_C_, Type				
No.	2	3	4	5
2	163.6, C	163.8, C	166.0, C	163.2, C
3	113.5, C	114.0, C	118.0, C	110.5, CH
4	181.6, C	181.4, C	180.3, C	176.0, C
5	152.7, C	156.5, C	156.4, C	151.8, C
6	95.2, CH	95.5, CH	99.5, CH	94.1, CH
7	153.3, C	157.8, C	159.7, C	151.2, C
8	125.6, C	127.8, C	127.6, C	127.8, C
9	144.4, C	148.7, C	149.4, C	147.1, C
10	103.0, C	103.0, C	101.8, C	107.9, C
2-CH_3_	18.4, CH_3_	18.5, CH_3_	18.0, CH_3_	19.3, CH_3_
3-CH_3_/CH_2_	8.8, CH_3_	8.8, CH_3_	52.2, CH_2_	
5-OCH_3_				56.4, CH_3_
7-OCH_3_	56.2, CH_3_	56.4, CH_3_		56.2, CH_3_
8-OCH_3_		60.9, CH_3_	60.5, CH_3_	

**Table 4:** Antibacterial activities of compounds **1**–**3** (MIC, μg/mL).

Strains	1	2	3	Chloramphenicol
Methicillin-resistant *Staphylococcus aureus*	32	-	64	4
*Micrococcus luteus*	32	-	-	4
*Pseudomonas aeruginosa*	64	-	-	0.5
*Vibrio harveyi*	32	-	-	2
*Vibrio vulnificus*	64	32	64	1

**Table 5:** DPPH radical scavenging activity of compounds **1**–**6** (IC_50_, μM).

**Compounds**	**1**	**2**	**3**	**4**
IC_50_ (μM)	24.10 ± 0.53	6.56 ± 0.21	>200 ± 3.10	>200 ± 2.75
**Compounds**	**5**	**6**	**BHT**	
IC_50_ (μM)	9.03 ± 0.65	>200 ± 1.80	61.39 ± 1.04	

## Data Availability

Not applicable.

## Supplementary Materials

The following Supplementary Materials are available online at https://www.mdpi.com/article/10.3390/md20080529/s1, Figures S1–S32: The analyzed data of MS, 1D, and 2D NMR spectra of compounds **1**–**5,** the analyzed data of MS, and 1D NMR spectra of compound **6,** and X-ray crystallographic file of compounds **2**, **4**, and **5**.

## References

[1] Lu L., Zheng Y.-Y., Shao C.-L., Wang C.-Y. (2019). Metabolites from marine invertebrates and their symbiotic microorganisms: Molecular diversity discovery, mining, and application. Mar. Life Sci. Technol..

[2] Jones W.J., Won Y.-J., Maas P., Smith P.J., Lutz R.A., Vrijenhoek R.C. (2006). Evolution of habitat use by deep-sea mussels. Mar. Biol..

[3] Hu X.-Y., Wang C.-Y., Li X.-M., Yang S.-Q., Li X., Wang B.-G., Si S.-Y., Meng L.-H. (2021). Cytochalasin derivatives from the endozoic *Curvularia verruculosa* CS-129, a fungus isolated from the deep-sea squat lobster *Shinkaia crosnieri* living in the cold seep environment. J. Nat. Prod..

[4] Shin H.J. (2020). Natural products from marine fungi. Mar. Drugs.

[5] Chi L.-P., Li X.-M., Wan Y.-P., Li X., Wang B.-G. (2020). Ophiobolin sesterterpenoids and farnesylated phthalide derivatives from the deep sea cold-seep-derived fungus *Aspergillus insuetus* SD-512. J. Nat. Prod..

[6] Chi L.-P., Li X.-M., Li L., Li X., Wang B.-G. (2020). Cytotoxic thiodiketopiperazine derivatives from the deep sea-derived fungus *Epicoccum nigrum* SD-388. Mar. Drugs.

[7] Yan L.-H., Li X.-M., Chi L.-P., Li X., Wang B.-G. (2022). Six new antimicrobial metabolites from the deep-sea sediment-derived fungus *Aspergillus fumigatus* SD-406. Mar. Drugs.

[8] Yan L.-H., Li P.-H., Li X.-M., Yang S.-Q., Liu K.-C., Wang B.-G., Li X. (2022). Chevalinulins A and B, proangiogenic alkaloids with a spiro[bicyclo[2.2.2]octane-diketopiperazine] skeleton from deep-sea cold-seep-derived fungus *Aspergillus chevalieri* CS-122. Org. Lett..

[9] Ngokpol S., Suwakulsiri W., Sureram S., Lirdprapamongkol K., Aree T., Wiyakrutta S., Mahidol C., Ruchirawat S., Kittakoop P. (2015). Drimane sesquiterpene-conjugated amino acids from a marine isolate of the fungus *Talaromyces minioluteus* (*Penicillium Minioluteum*). Mar. Drugs.

[10] Tanahashi T., Takenaka Y., Nagakura N., Hamada N. (2000). 2,3-Dialkylchromones from mycobiont cultures of the lichen *Graphis scripta*. Heterocycles.

[11] Wu M.-C., Peng C.-F., Chen I.-S., Tsai I.-L. (2011). Antitubercular chromones and flavonoids from *Pisonia aculeata*. J. Nat. Prod..

[12] Wang S., Li X.-M., Teuscher F., Li D.-L., Diesel A., Ebel R., Proksch P., Wang B.-G. (2006). Chaetopyranin, a benzaldehyde derivative, and other related metabolites from *Chaetomium globosum*, an endophytic fungus derived from the marine red alga *Polysiphonia urceolata*. J. Nat. Prod..

[13] Crystallographic Data of Compounds **2**, **4**, and **5** Have Been Deposited in the Cambridge Crystallographic Data Centre as CCDCs 2153737 (for **2**), 2153739 (for **4**), and 2153738 (for **5**). CCDC, 12 Union Road, Cambridge CB21EZ, UK. http://www.ccdc.cam.ac.uk/data_request/cif.

[14] Sheldrick G.M. (1996). SADABS, Software for Empirical Absorption Correction.

[15] Sheldrick G.M. (1997). SHELXTL, Structure Determination Software Programs.

[16] Sheldrick G.M. (1997). SHELXL-97 and SHELXS-97, Program for X-ray Crystal Structure Solution and Refinement.

[17] Pierce C.G., Uppuluri P., Tristan A.R., Wormley F.L., Mowat E., Ramage G., Lopez-Ribot J.L. (2008). A simple and reproducible 96-well plate-based method for the formation of fungal biofilms and its application to antifungal susceptibility testing. Nat. Protoc..

[18] Sharma O.P., Bhat T.K. (2009). DPPH antioxidant assay revisited. Food Chem..

[19] Zhang P., Li X.-M., Wang J.-N., Li X., Wang B.-G. (2015). New butenolide derivatives from the marine-derived fungus *Paecilomyces variotii* with DPPH radical scavenging activity. Phytochem. Lett..

