# Supplementary material for: Aromatic Polyketides from the Deep-Sea Cold-Seep Mussel Associated Endozoic Fungus Talaromyces minioluteus CS-138
Source: Mar Drugs. 2022 Aug 18;20(8):529. doi: 10.3390/md20080529 (PMC9409973; doi:10.3390/md20080529)

Supplementary Material

Aromatic Polyketides from the Deep-Sea Cold-Seep Mussel Associated Endozoic Fungus *Talaromyces minioluteus* CS-138

Qi Song [1,2,3 ǀ], Sui-Qun Yang [1,2,4 ǀ], Xiao-Ming Li [1,2,4], Xue-Yi Hu [1,2,3], Xin Li [1,2,4] and Bin-Gui Wang [1,2,3,4,*]

[1] CAS and Shandong Province Key Laboratory of Experimental Marine Biology, Institute of Oceanology, Chinese Academy of Sciences, Nanhai Road 7, Qingdao 266071, China
[2] Laboratory of Marine Biology and Biotechnology, Qingdao National Laboratory for Marine Science and Technology, Wenhai Road 1, Qingdao 266237, China
[3] College of Marine Science, University of Chinese Academy of Sciences, Yuquan Road 19A, Beijing 100049, China
[4] Center for Ocean Mega-Science, Chinese Academy of Sciences, Nanhai Road 7, Qingdao 266071, China
* Correspondence: wangbg@ms.qdio.ac.cn (B.-G.W.); Tel.: +86-532-8289-8553 (B.-G.W.)
ǀ These authors contributed equally to this work.

Contents

Figure S1. HRESIMS spectrum of compound **1**;

Figure S2. ^1H NMR (500 MHz, DMSO-d_6) spectrum of compound **1**;

Figure S3. ^{13}C NMR (125 MHz, DMSO-d_6) and DEPT spectra of compound **1**;

Figure S4. COSY spectrum of compound **1**;

Figure S5. HSQC spectrum of compound **1**;

Figure S6. HMBC spectrum of compound **1**;

Figure S7. HRESIMS spectrum of compound **2**;

Figure S8. ^1H NMR (500 MHz, DMSO-d_6) spectrum of compound **2**;

Figure S9. ^{13}C NMR (125 MHz, DMSO-d_6) and DEPT spectra of compound **2**;

Figure S10. HSQC spectrum of compound **2**;

Figure S11. HMBC spectrum of compound **2**;

Figure S12. Crystal packing of compound **2** at 293(2) K;

Figure S13. HRESIMS spectrum of compound **3**;

Figure S14. ^1H NMR (500 MHz, DMSO-d_6) spectrum of compound **3**;

Figure S15. ^{13}C NMR (125 MHz, DMSO-d_6) and DEPT spectra of compound **3**;

Figure S16. HSQC spectrum of compound **3**;

Figure S17. HMBC spectrum of compound **3**;

Figure S18. HRESIMS spectrum of compound **4**;

Figure S19. ^1H NMR (500 MHz, DMSO-d_6) spectrum of compound **4**;

Figure S20. ^{13}C NMR (125 MHz, DMSO-d_6) and DEPT spectra of compound **4**;

Figure S21. HSQC spectrum of compound **4**;

Figure S22. HMBC spectrum of compound **4**;

Figure S23. Crystal packing of compound **4** at 296(2) K;

Figure S24. HRESIMS spectrum of compound **5**;

Figure S25. ^1H NMR (500 MHz, DMSO-d_6) spectrum of compound **5**;

Figure S26. ^{13}C NMR (125 MHz, DMSO-d_6) and DEPT spectra of compound **5**;

Figure S27. HSQC spectrum of compound **5**;

Figure S28. HMBC spectrum of compound **5**;

Figure S29. Crystal packing of compound **5** at 298(2) K;

Figure S30. HRESIMS spectrum of compound **6**;

Figure S31. ^1H NMR (500 MHz, DMSO-d_6) spectrum of compound **6**;

Figure S32. ^{13}C NMR (125 MHz, DMSO-d_6) and DEPT spectra of compound **6**.

Figure S1. HRESIMS spectrum of compound **1**.

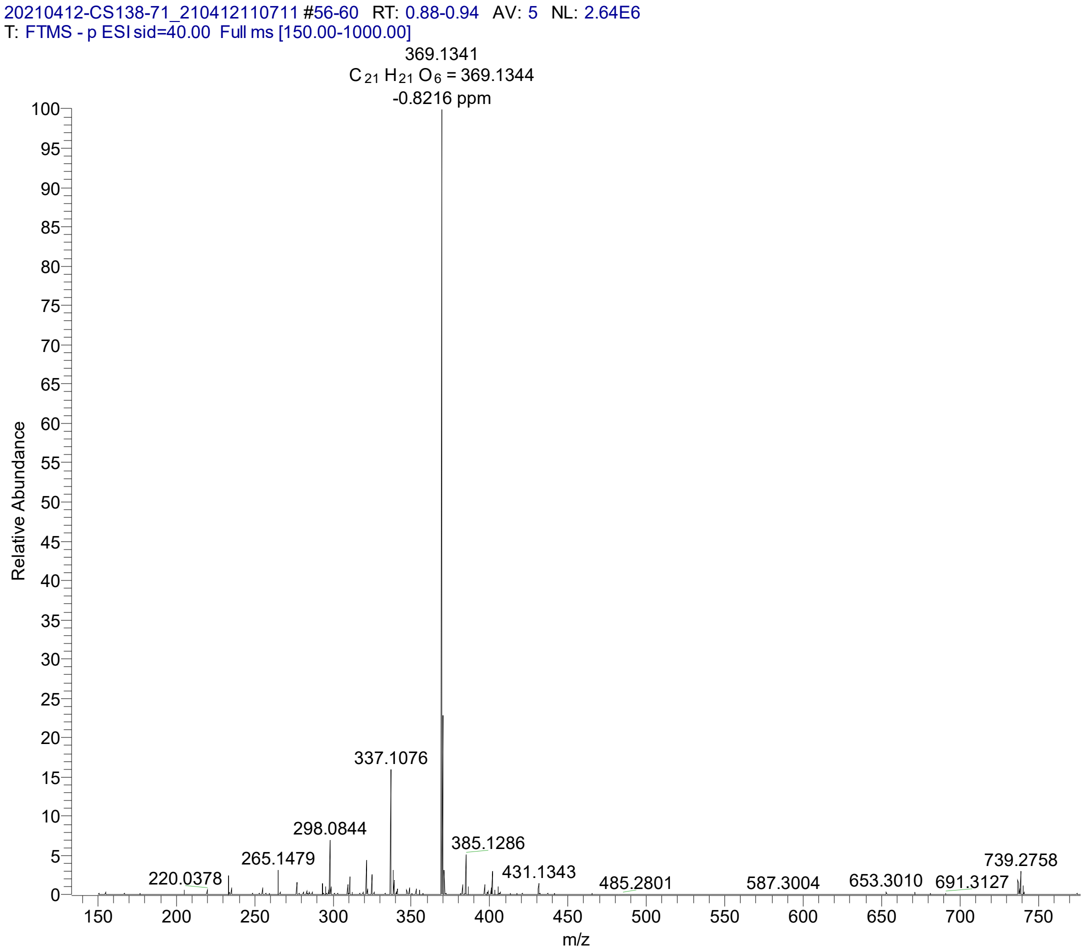

Figure S2. ^{1}H NMR (500 MHz, DMSO-d_6) spectrum of compound **1**.

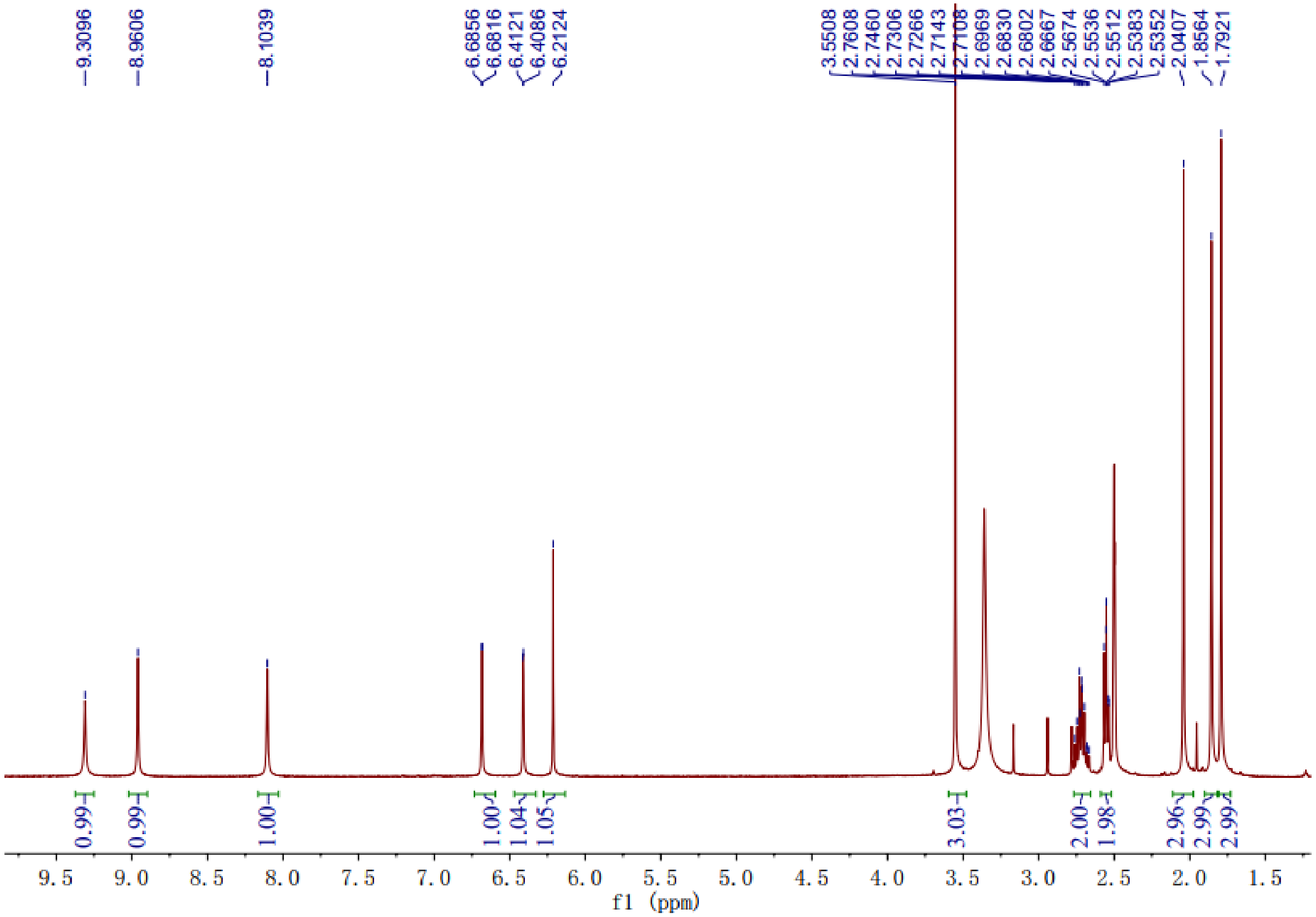

Figure S3. ^{13}C NMR (125 MHz, DMSO-d_6) and DEPT spectra of compound **1**.

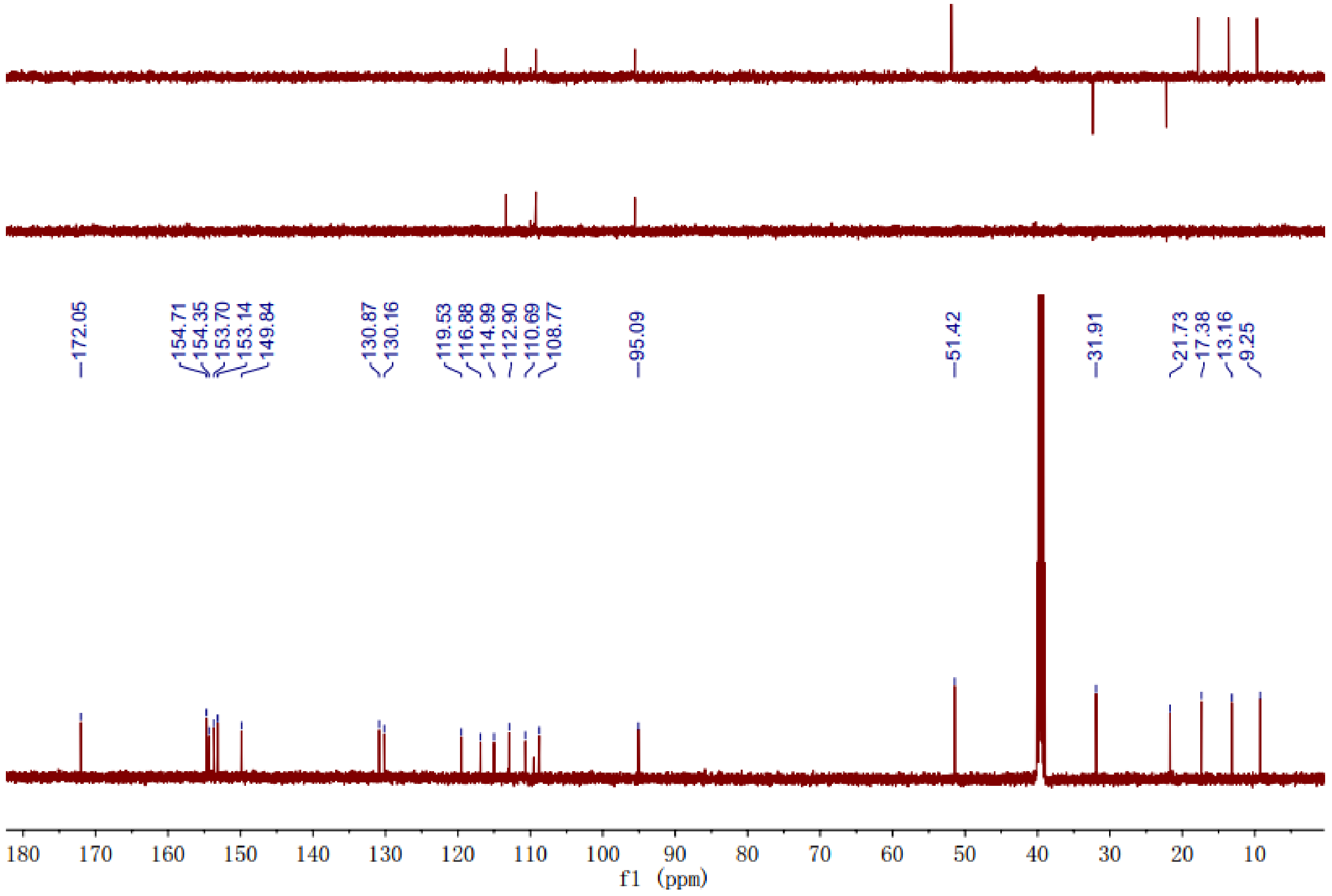

Figure S4. COSY spectrum of compound **1**.

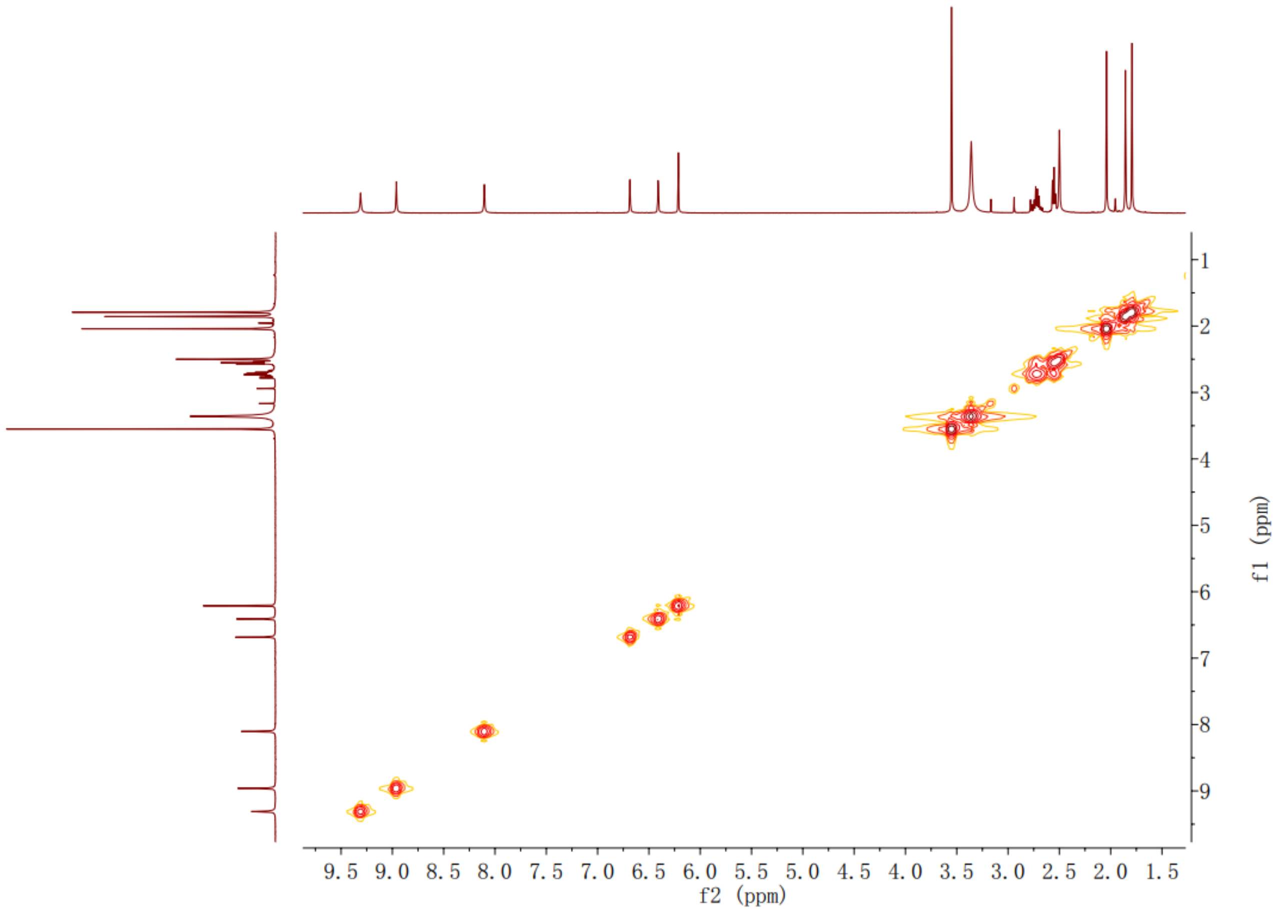

Figure S5. HSQC spectrum of compound **1**.

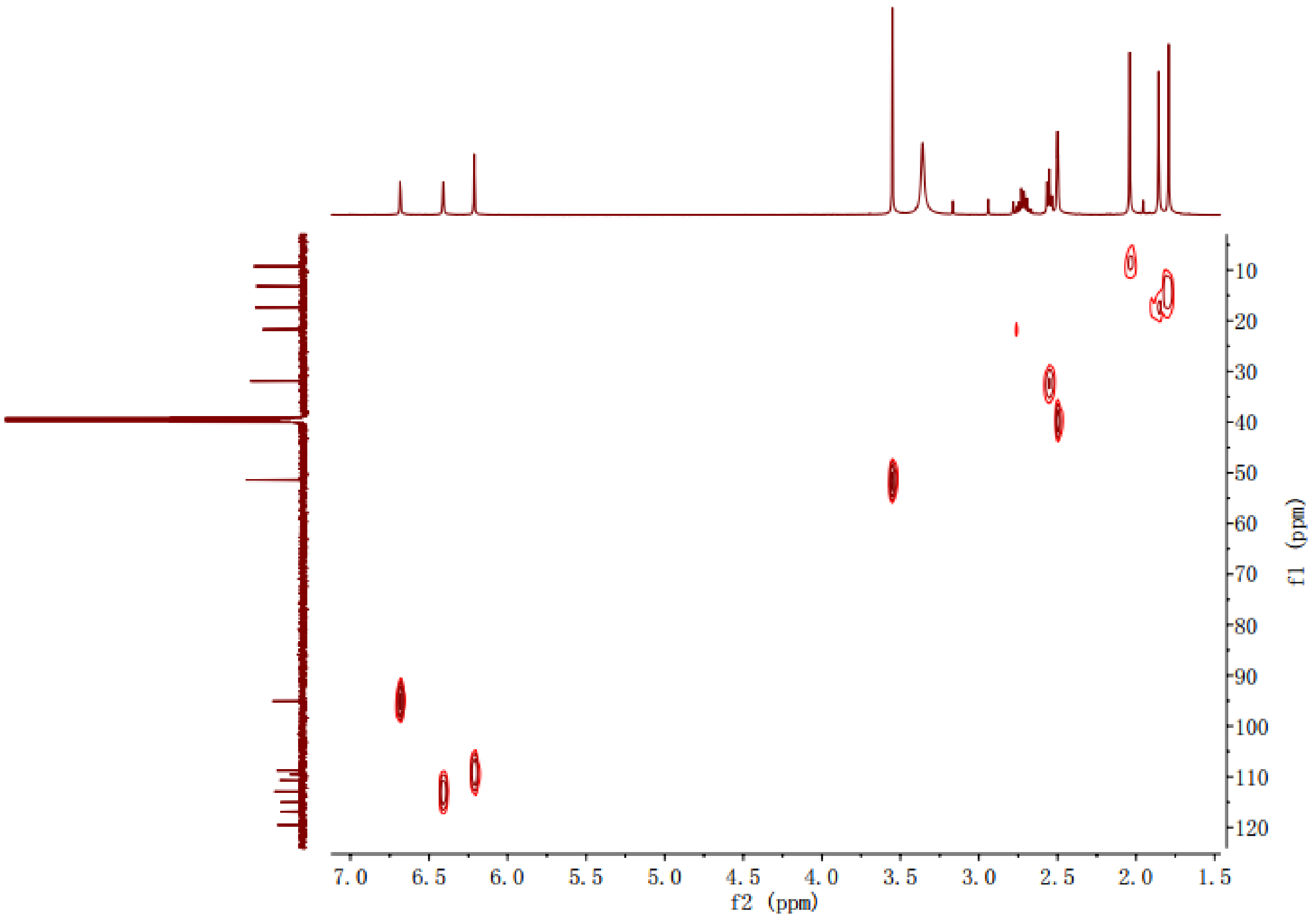

Figure S6. HMBC spectrum of compound **1**.

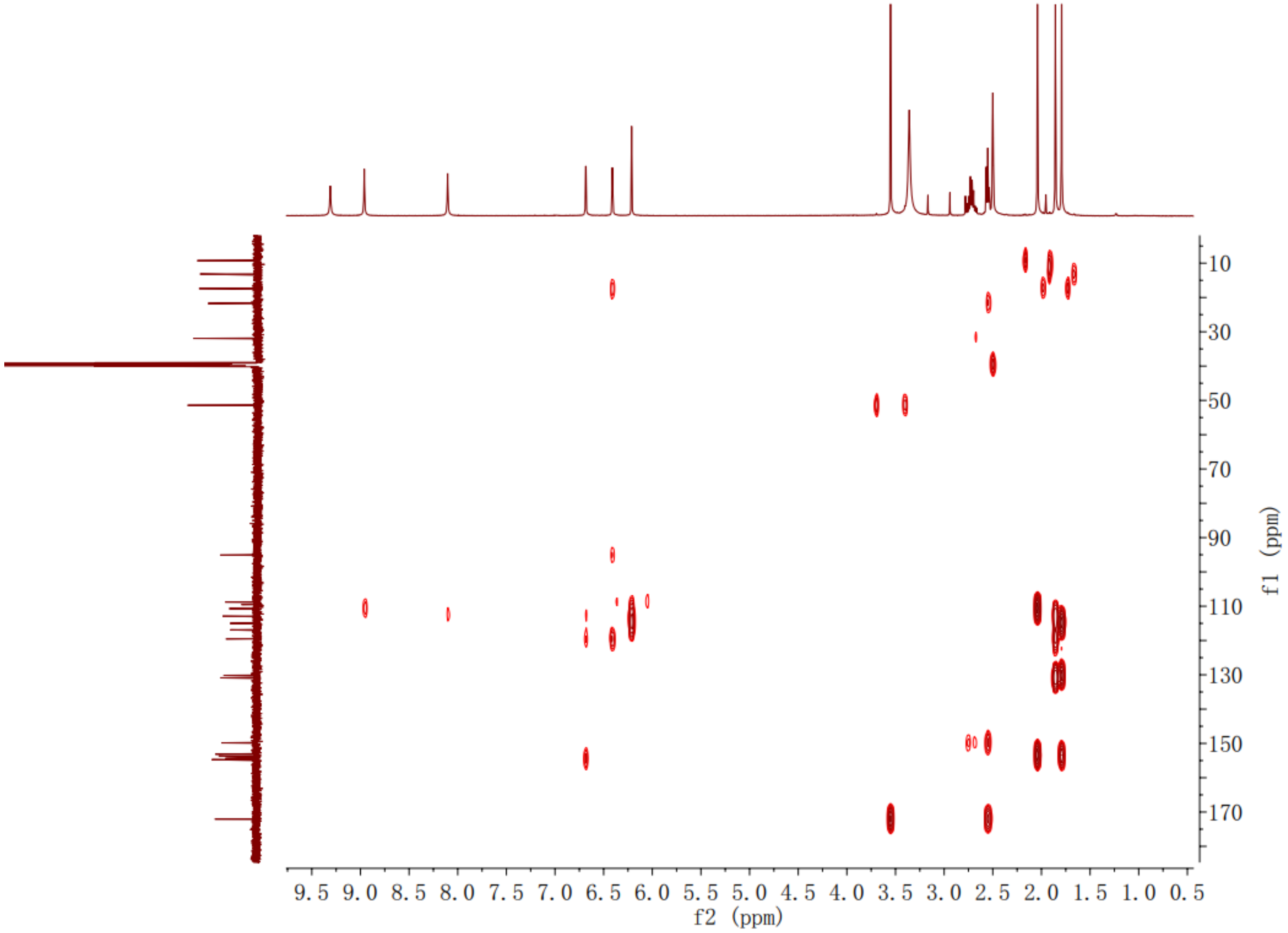

Figure S7. HRESIMS spectrum of compound **2**.

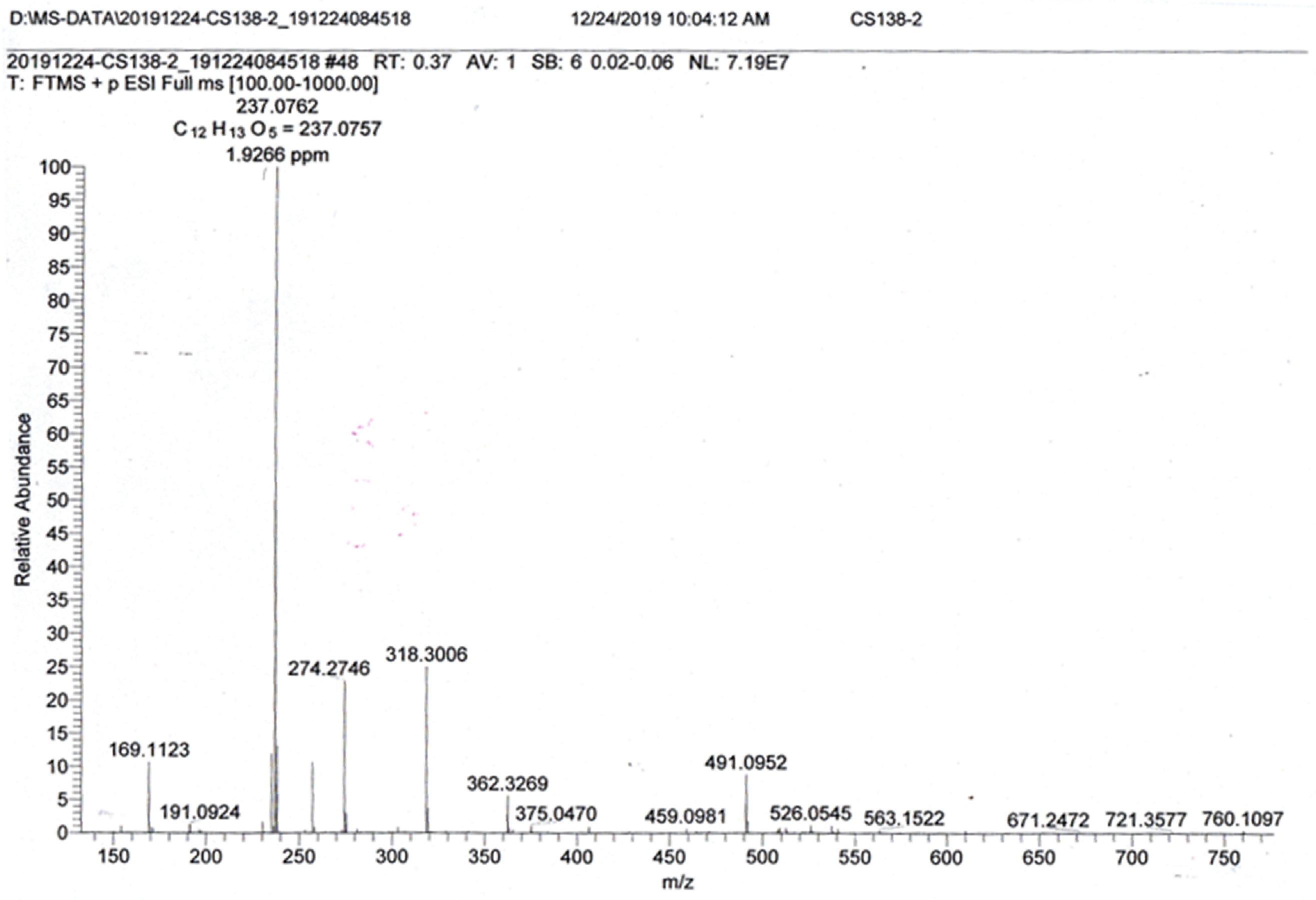

Figure S8. ^{1}H NMR (500 MHz, DMSO-d_6) spectrum of compound **2**.

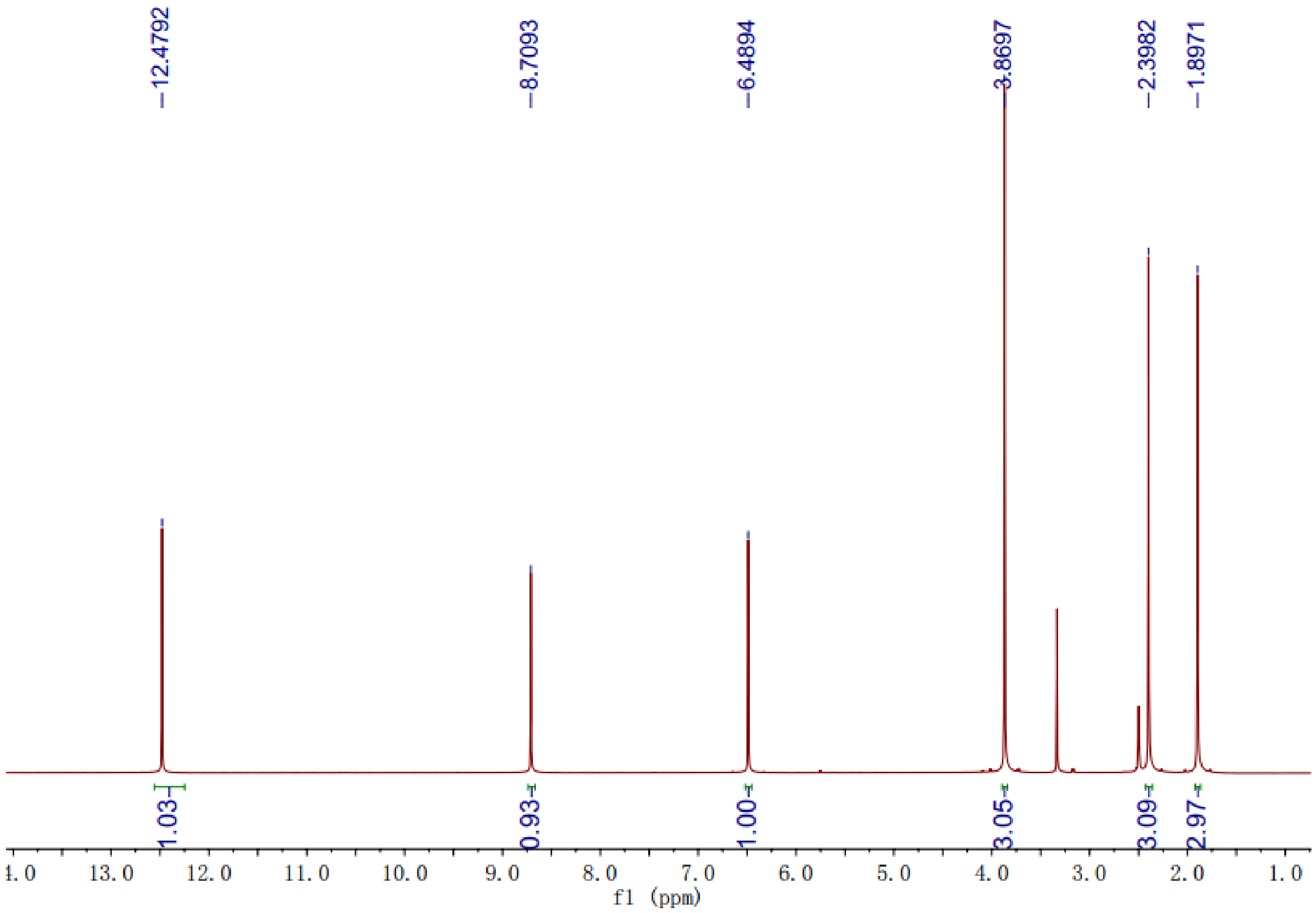

Figure S9. ^{13}C NMR (125 MHz, DMSO-d_6) and DEPT spectra of compound **2**.

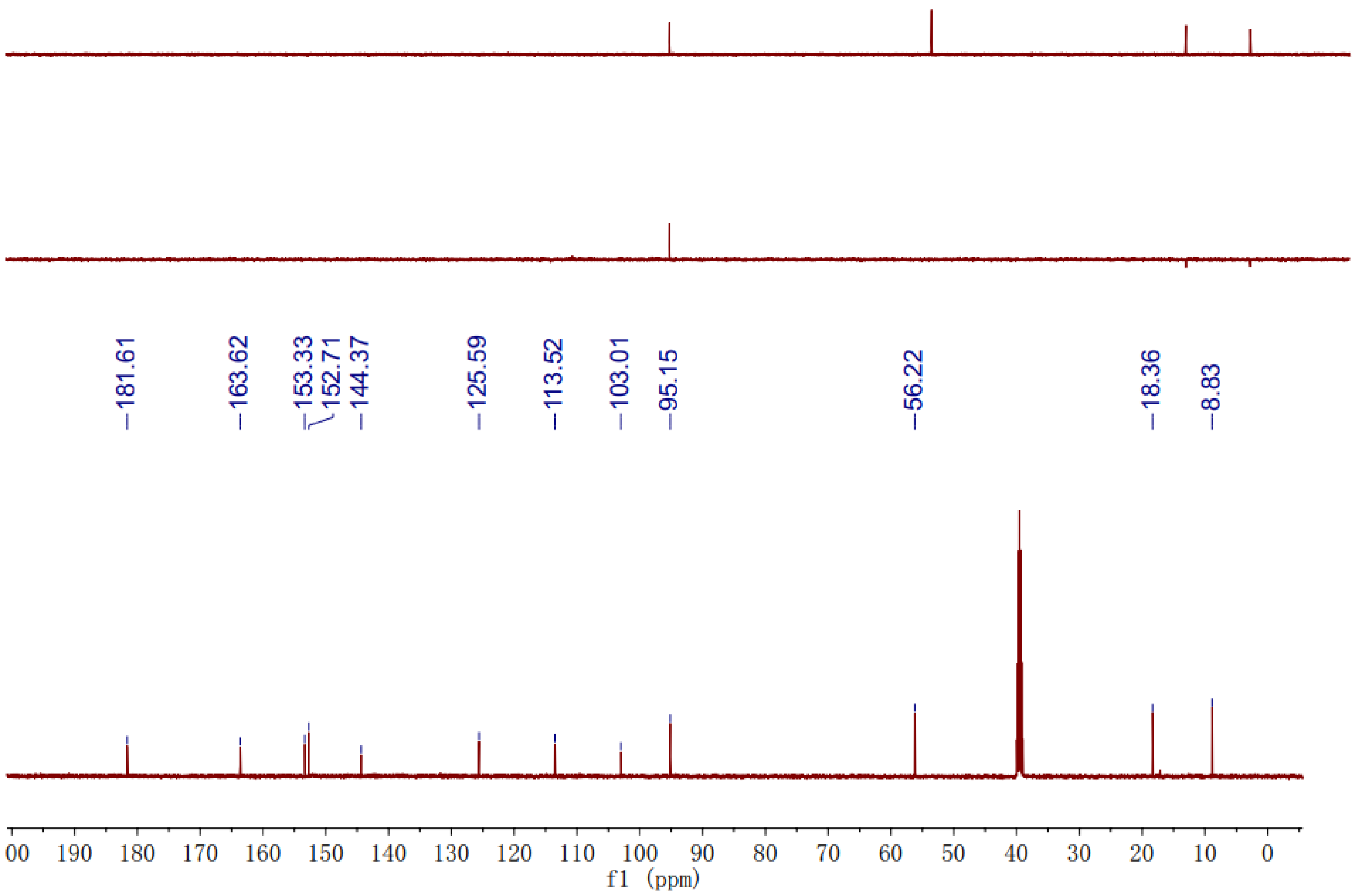

Figure S10. HSQC spectrum of compound **2**.

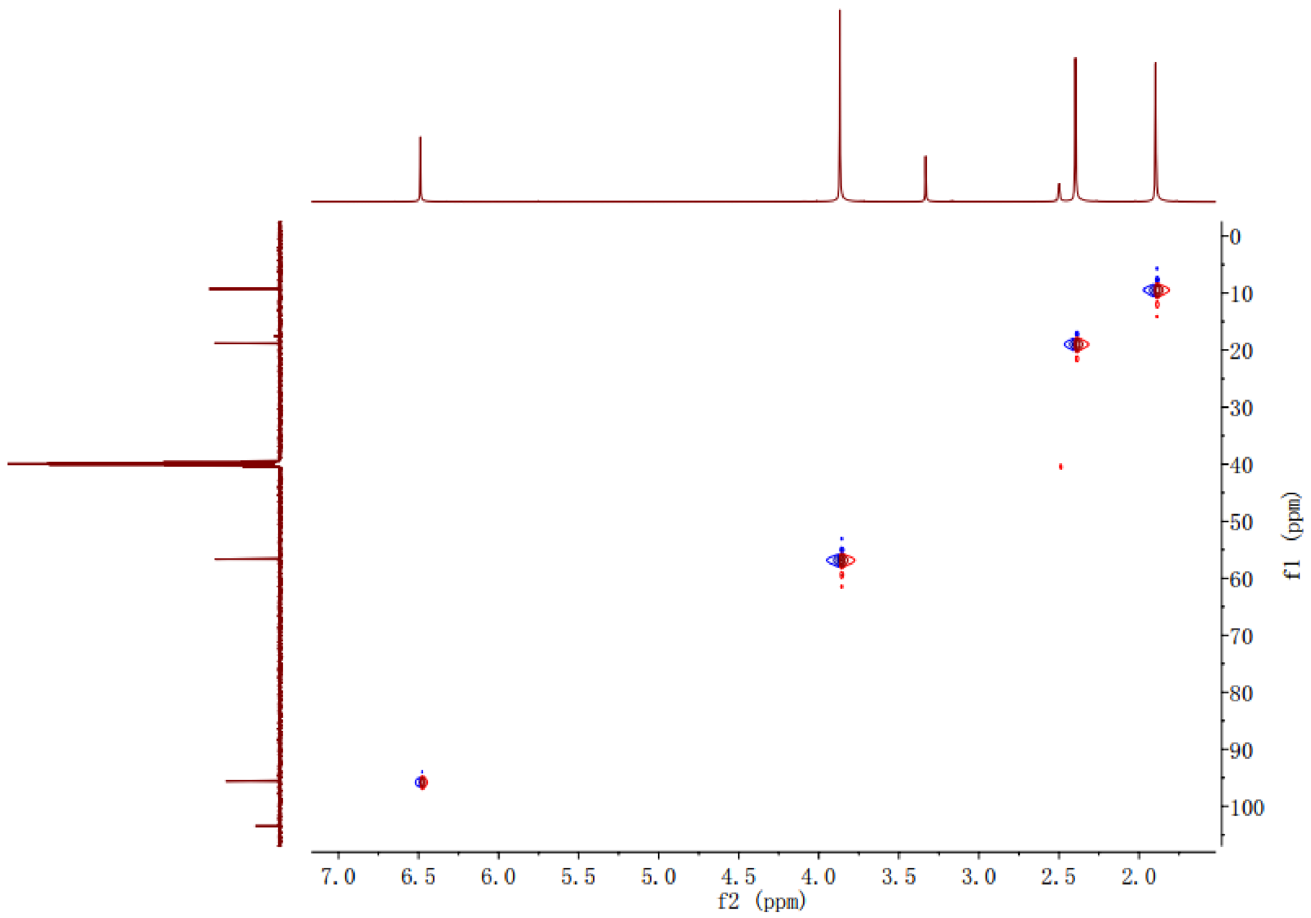

Figure S11. HMBC spectrum of compound **2**.

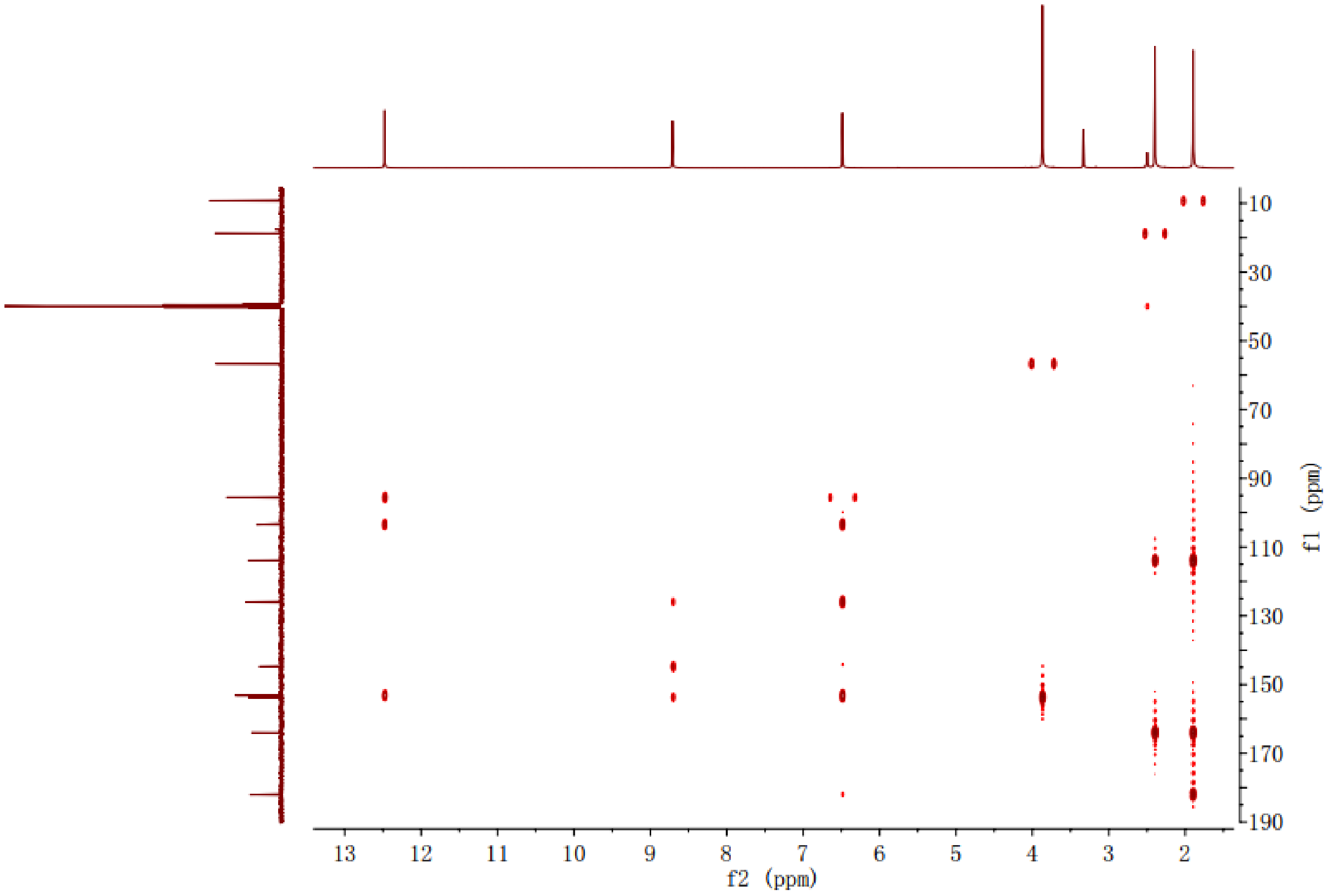

Figure S12. Crystal packing of compound **2** at 293(2) K.

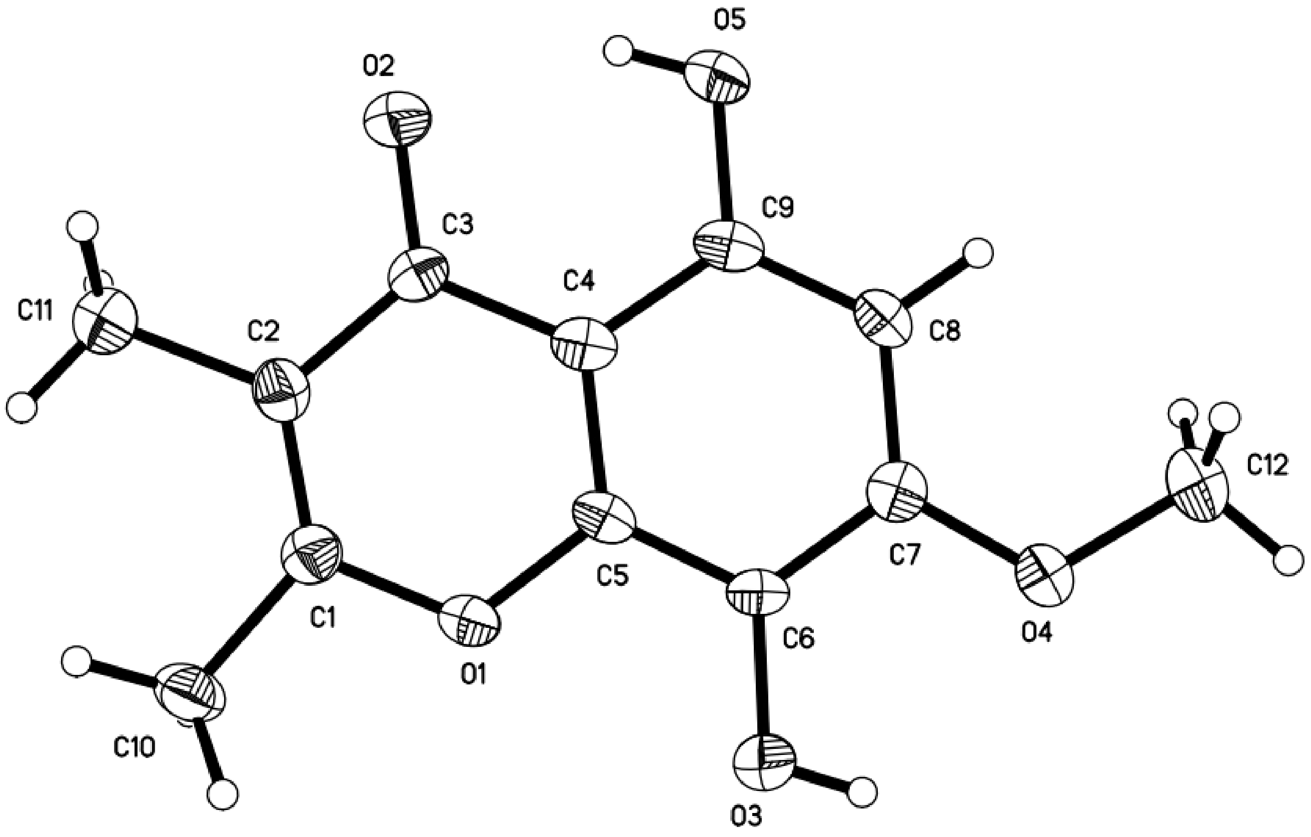

Figure S13. HRESIMS spectrum of compound **3**.

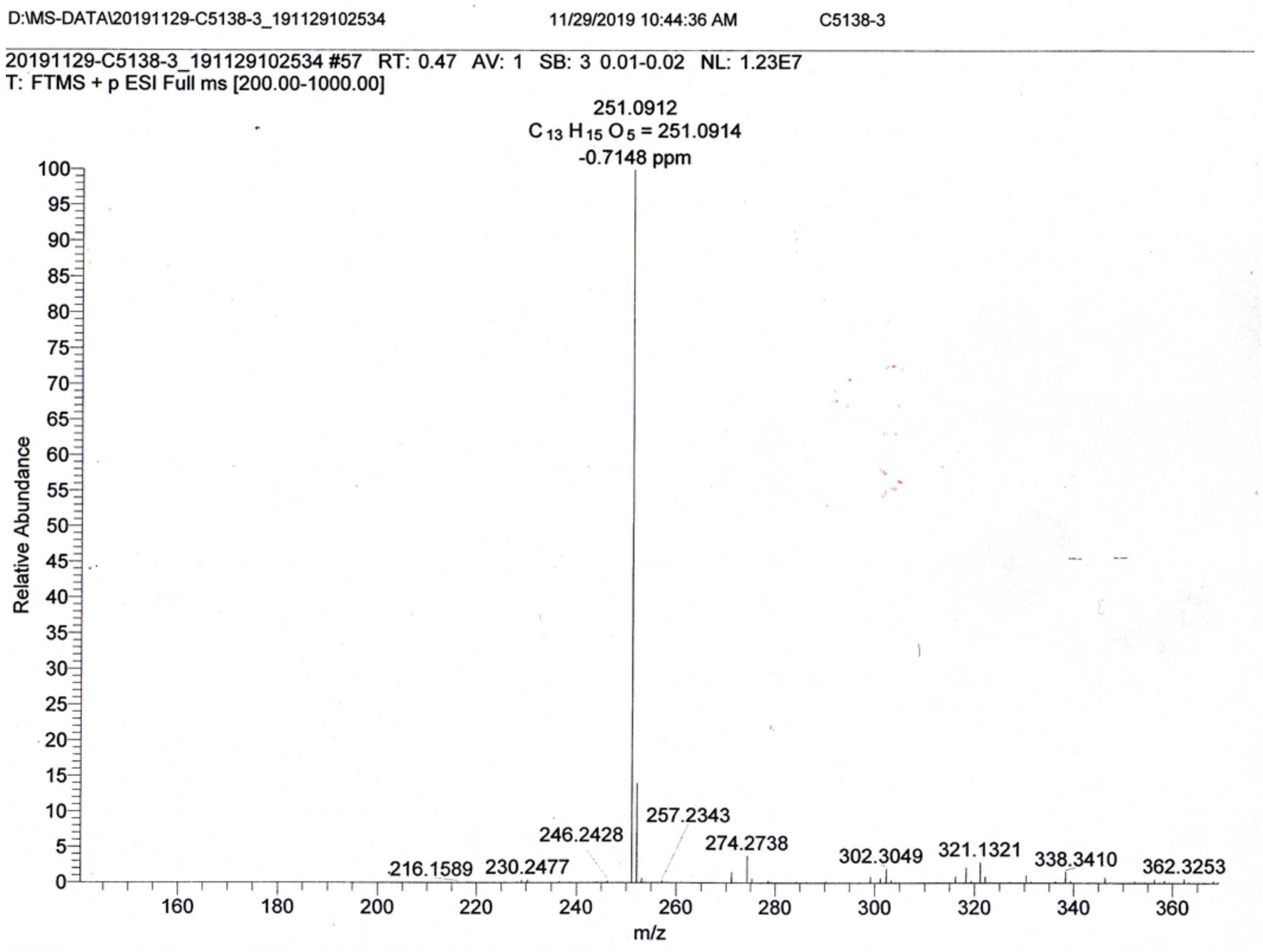

Figure S14. ^{1}H NMR (500 MHz, DMSO-d_6) spectrum of compound **3**.

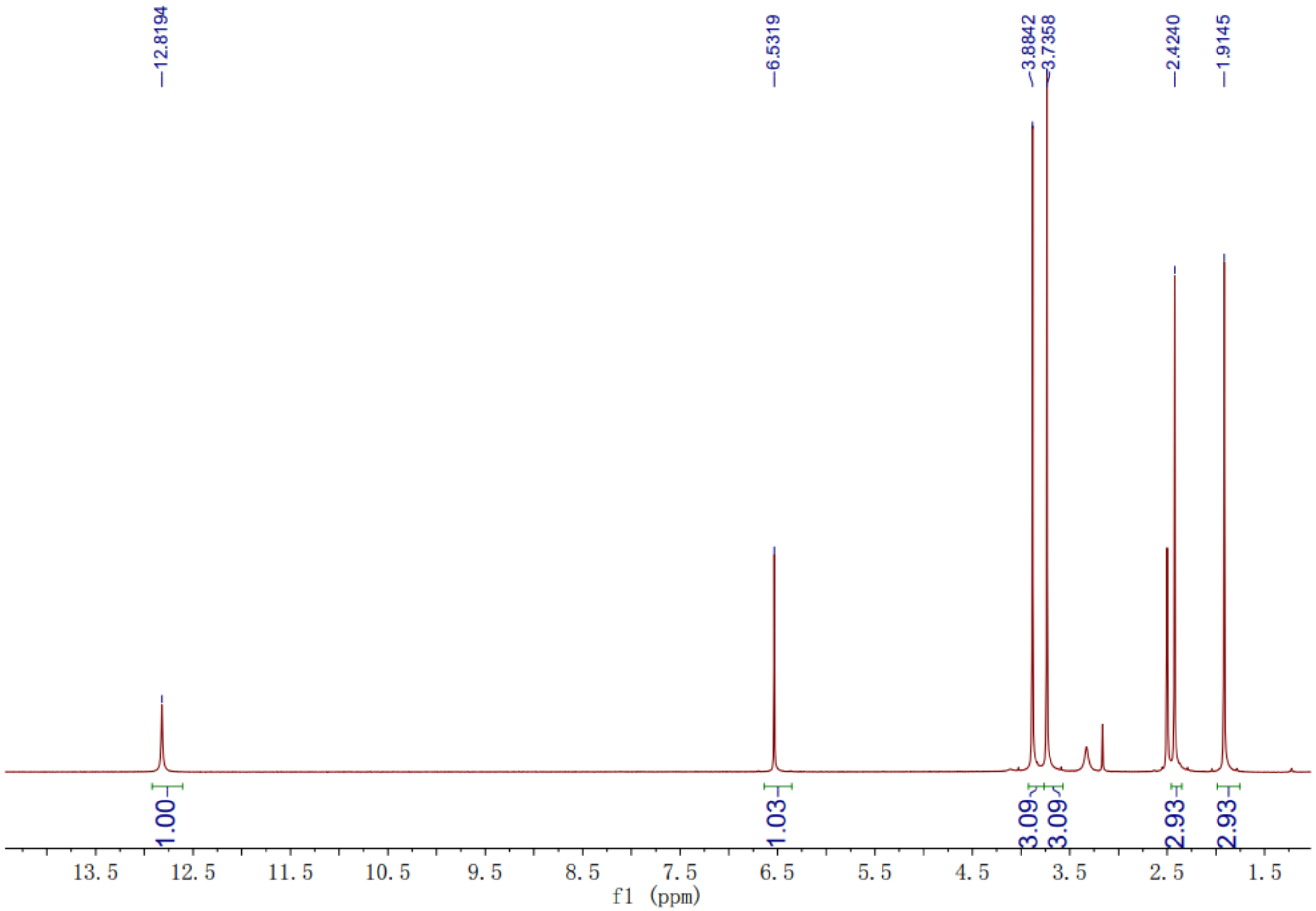

Figure S15. ^{13}C NMR (125 MHz, DMSO-d_6) and DEPT spectra of compound **3**.

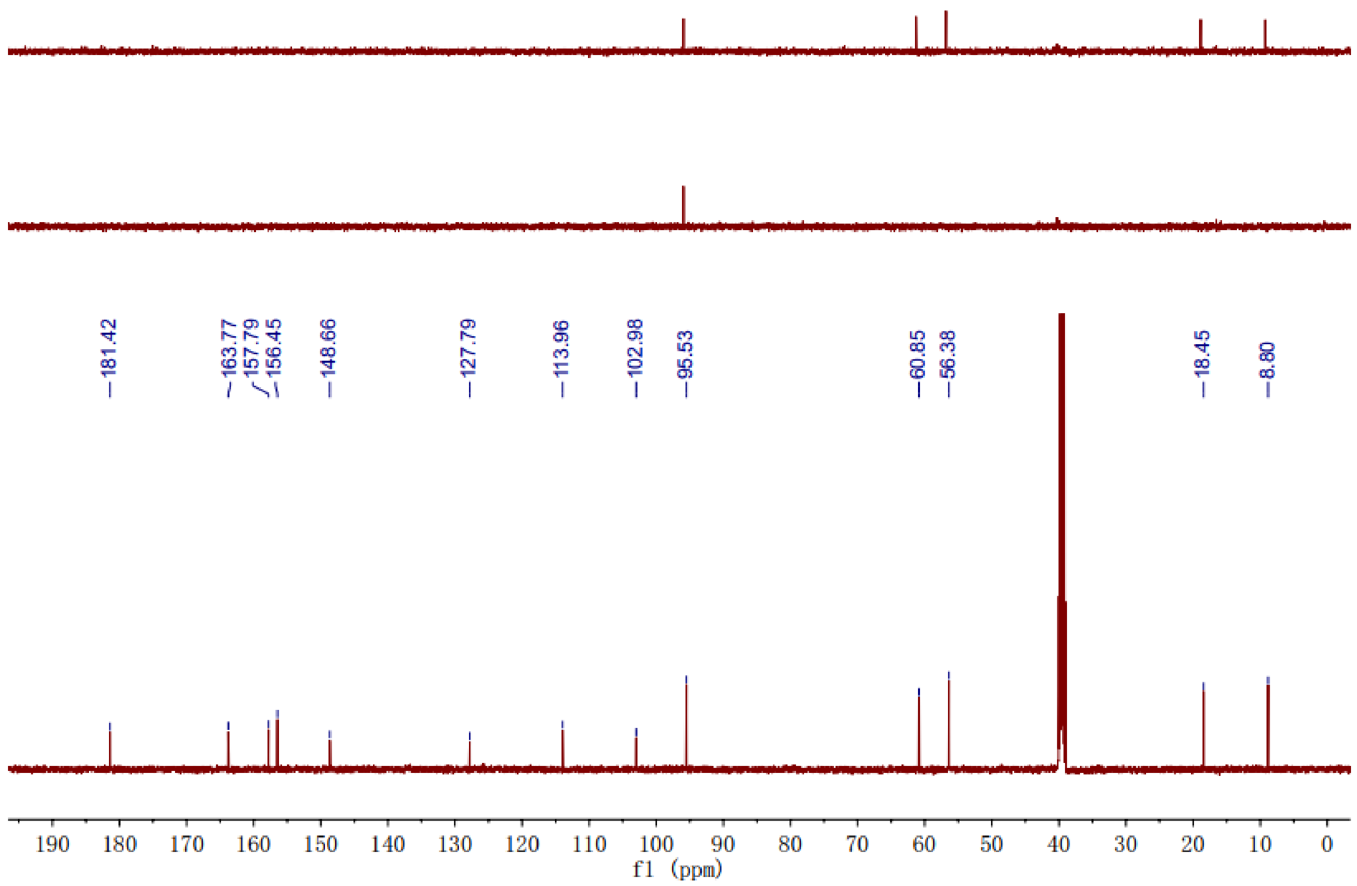

Figure S16. HSQC spectrum of compound **3**.

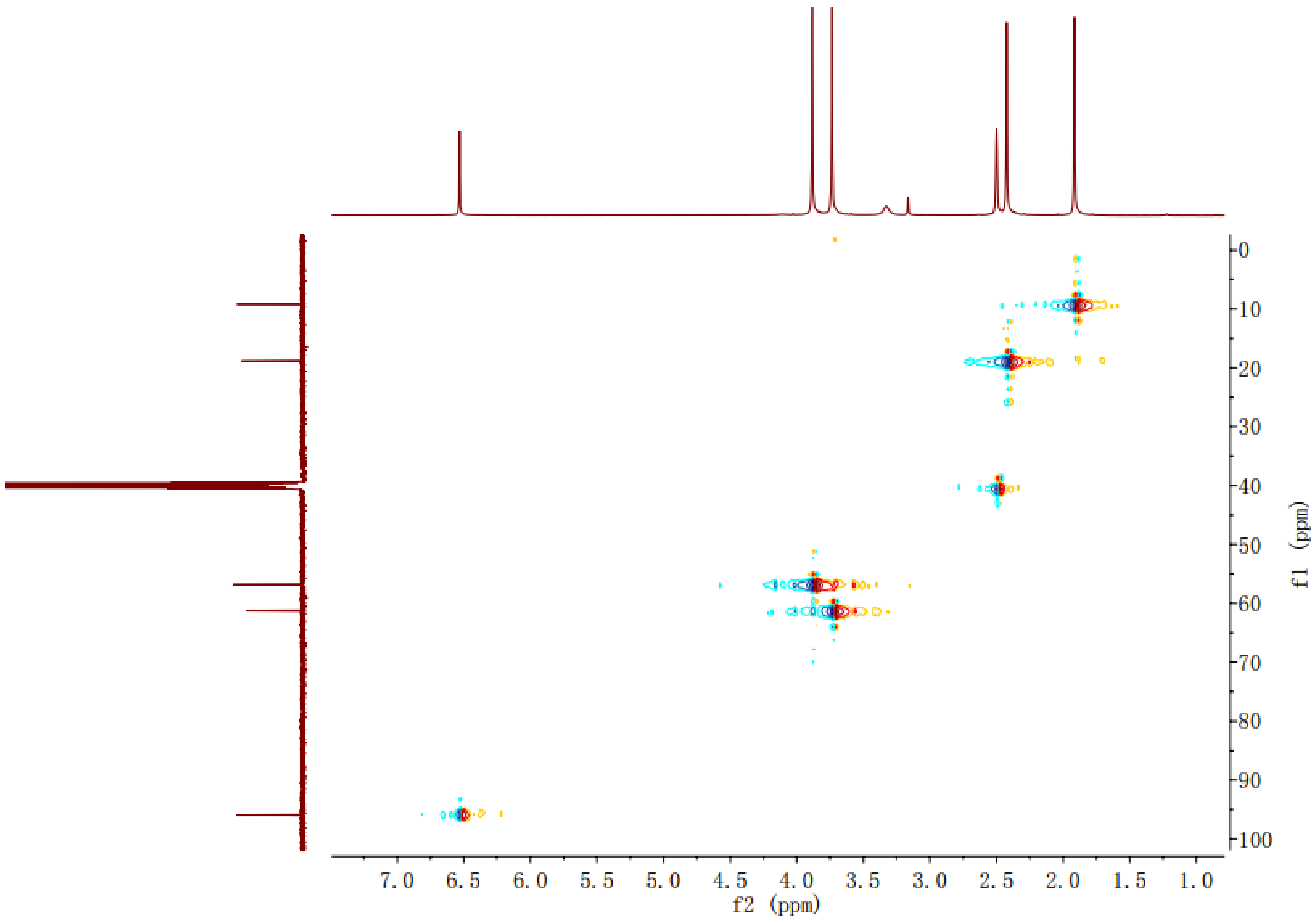

Figure S17. HMBC spectrum of compound **3**.

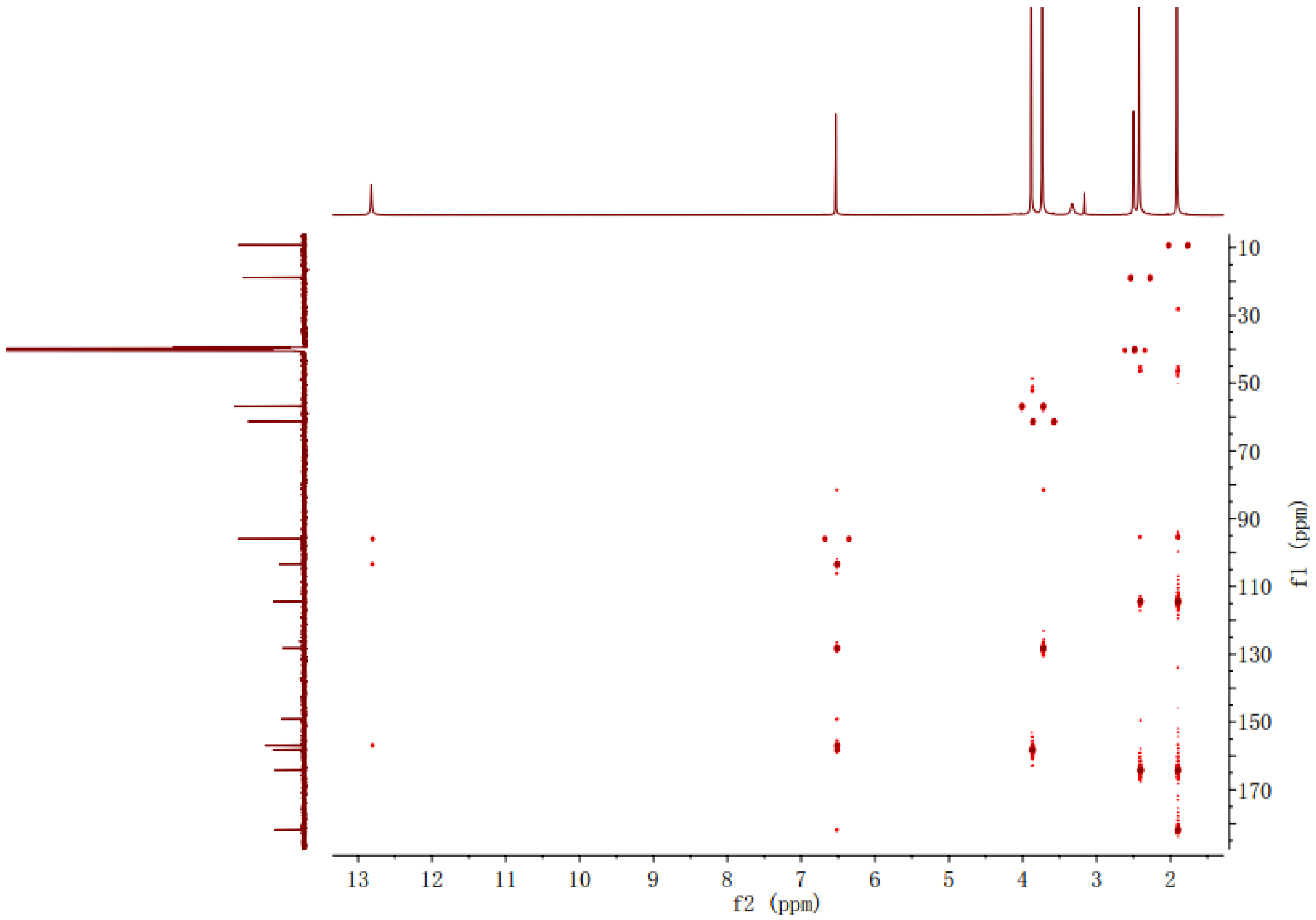

Figure S18. HRESIMS spectrum of compound **4**.

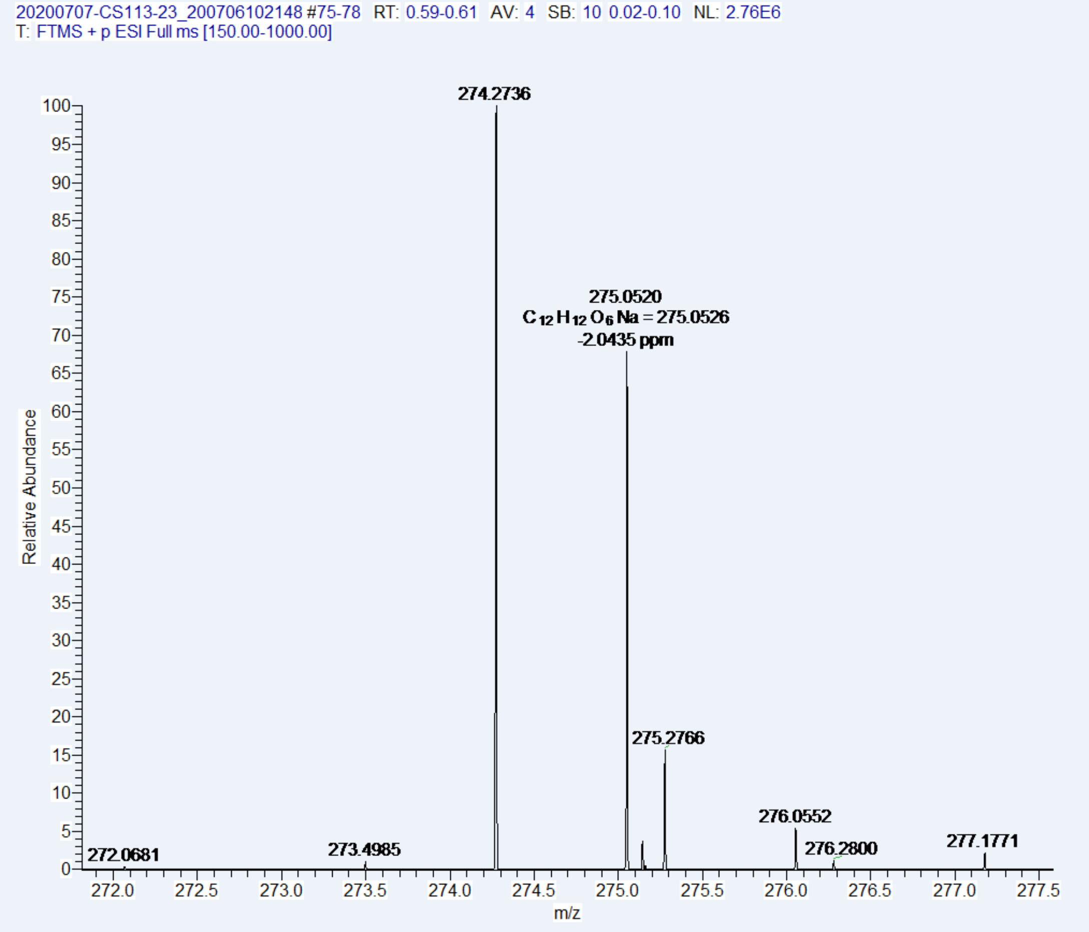

Figure S19. ^{1}H NMR (500 MHz, DMSO-d_6) spectrum of compound **4**.

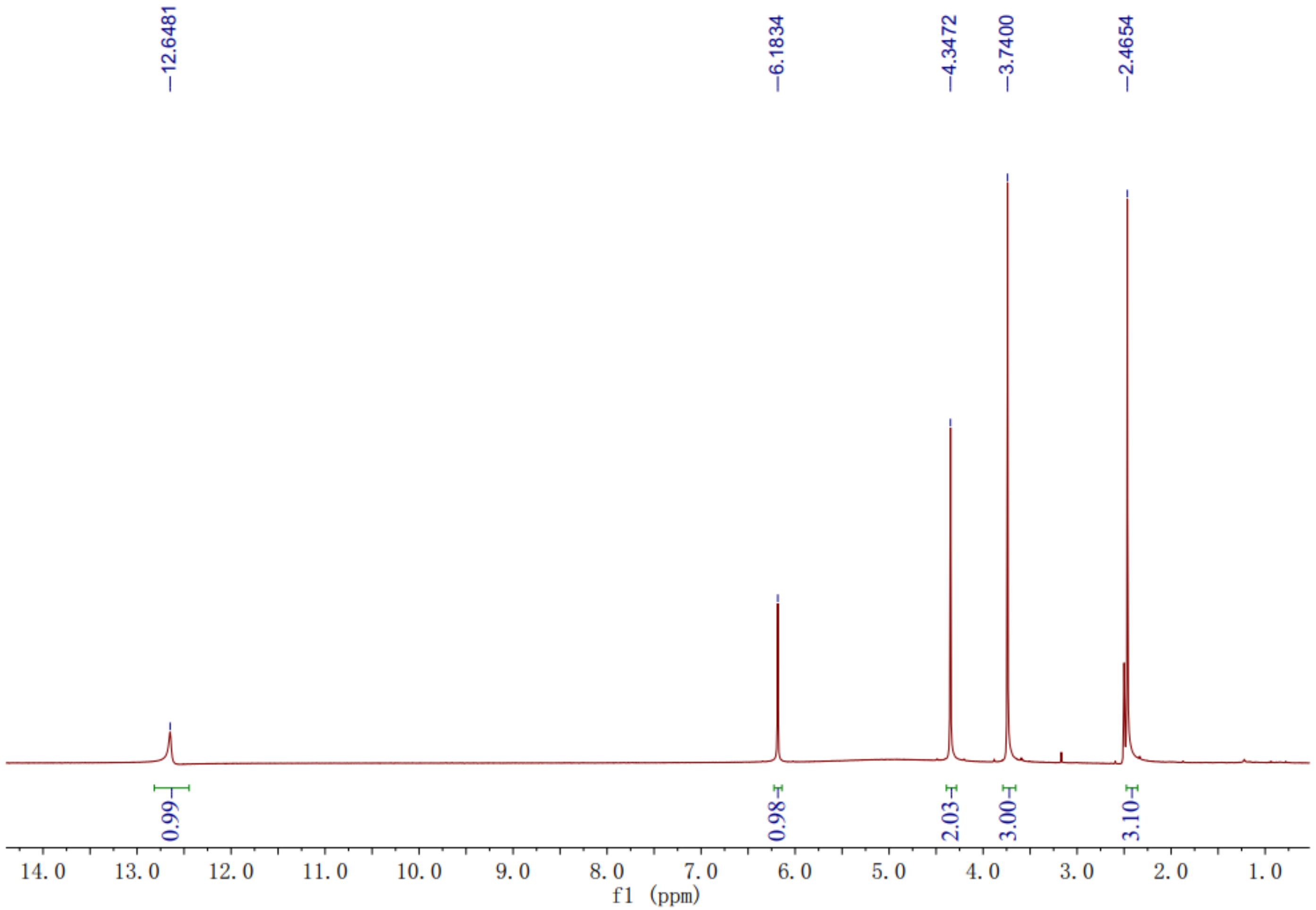

Figure S20. ^{13}C NMR (125 MHz, DMSO-d_6) and DEPT spectra of compound **4**.

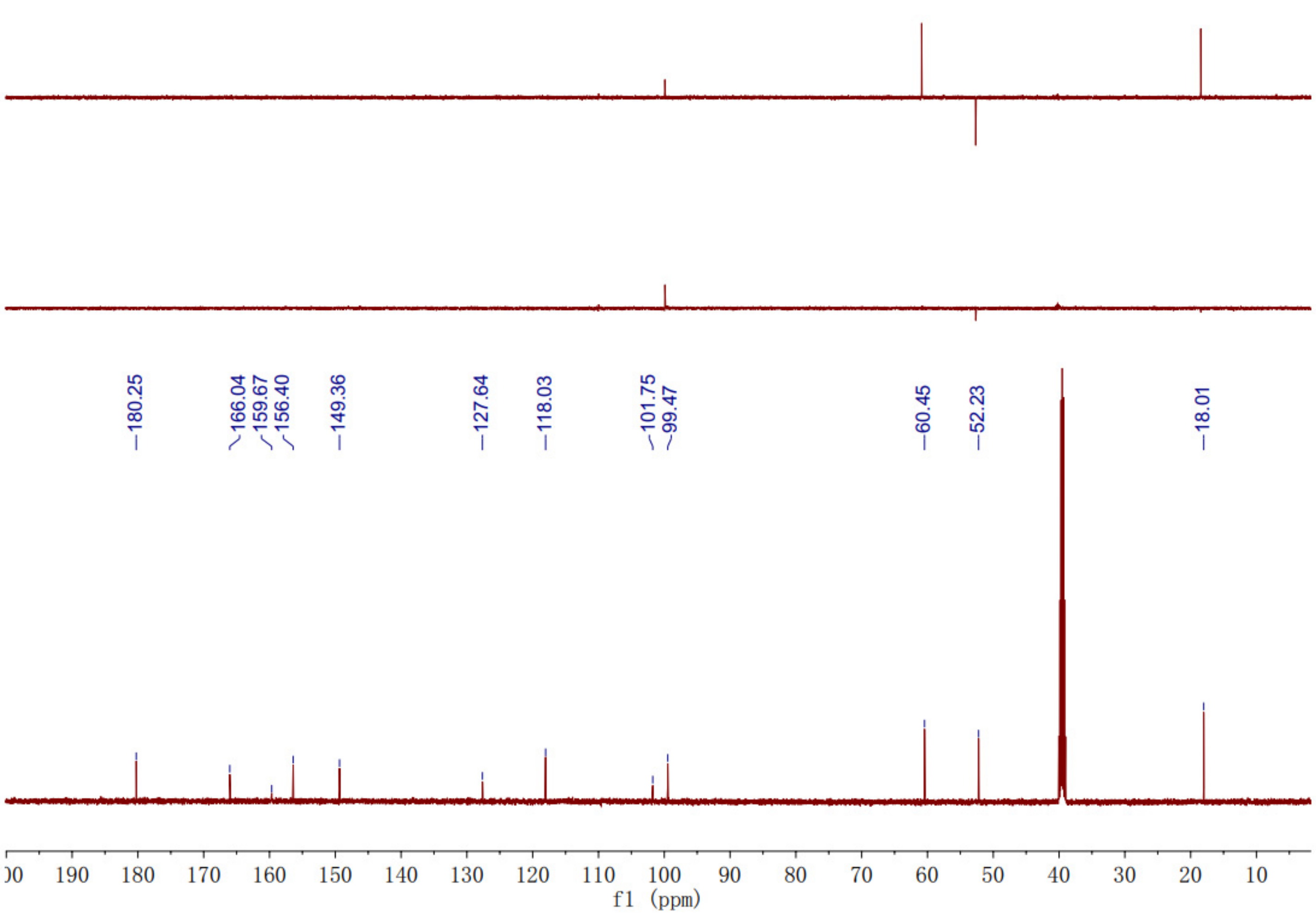

Figure S21. HSQC spectrum of compound **4**.

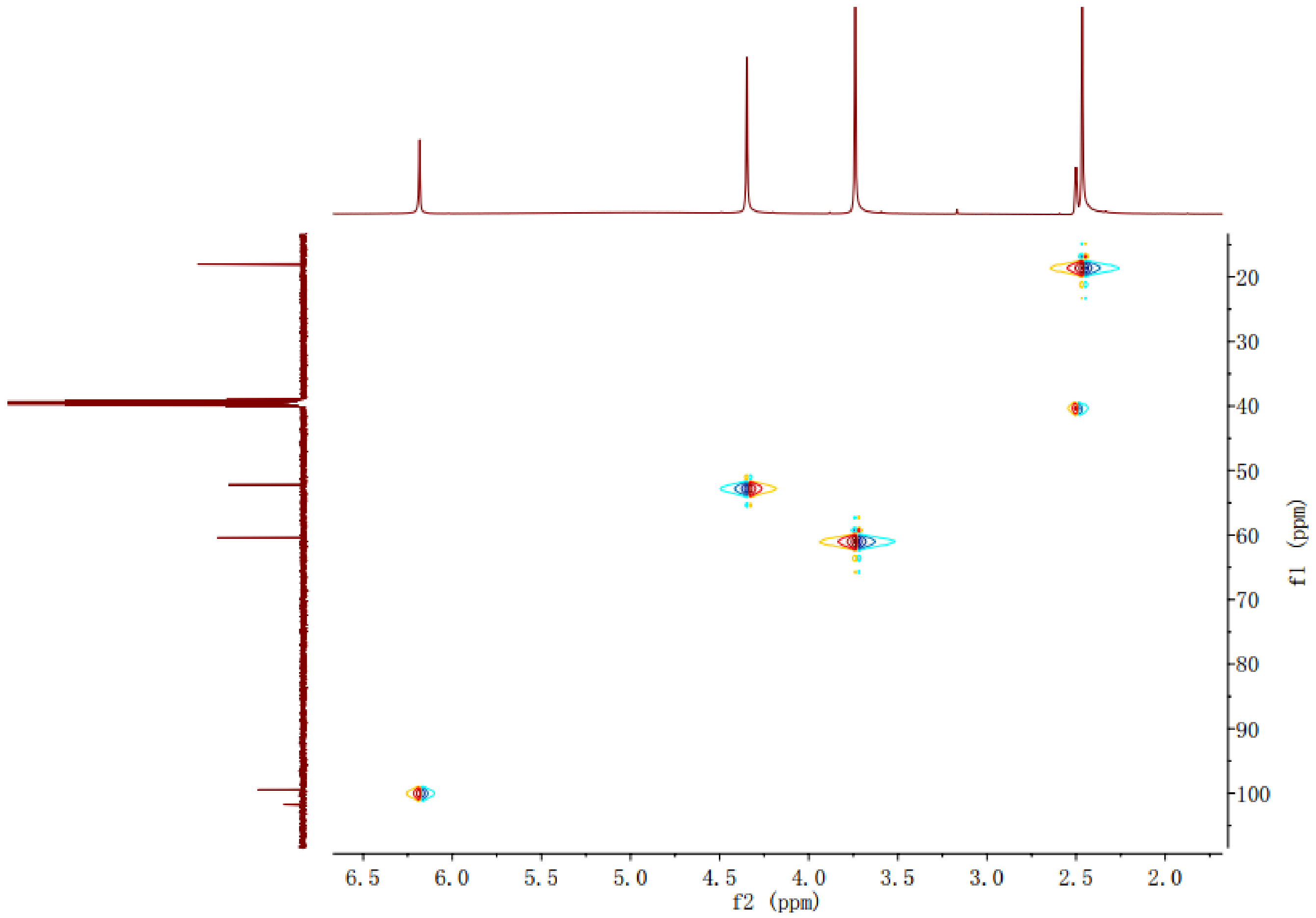

Figure S22. HMBC spectrum of compound **4**.

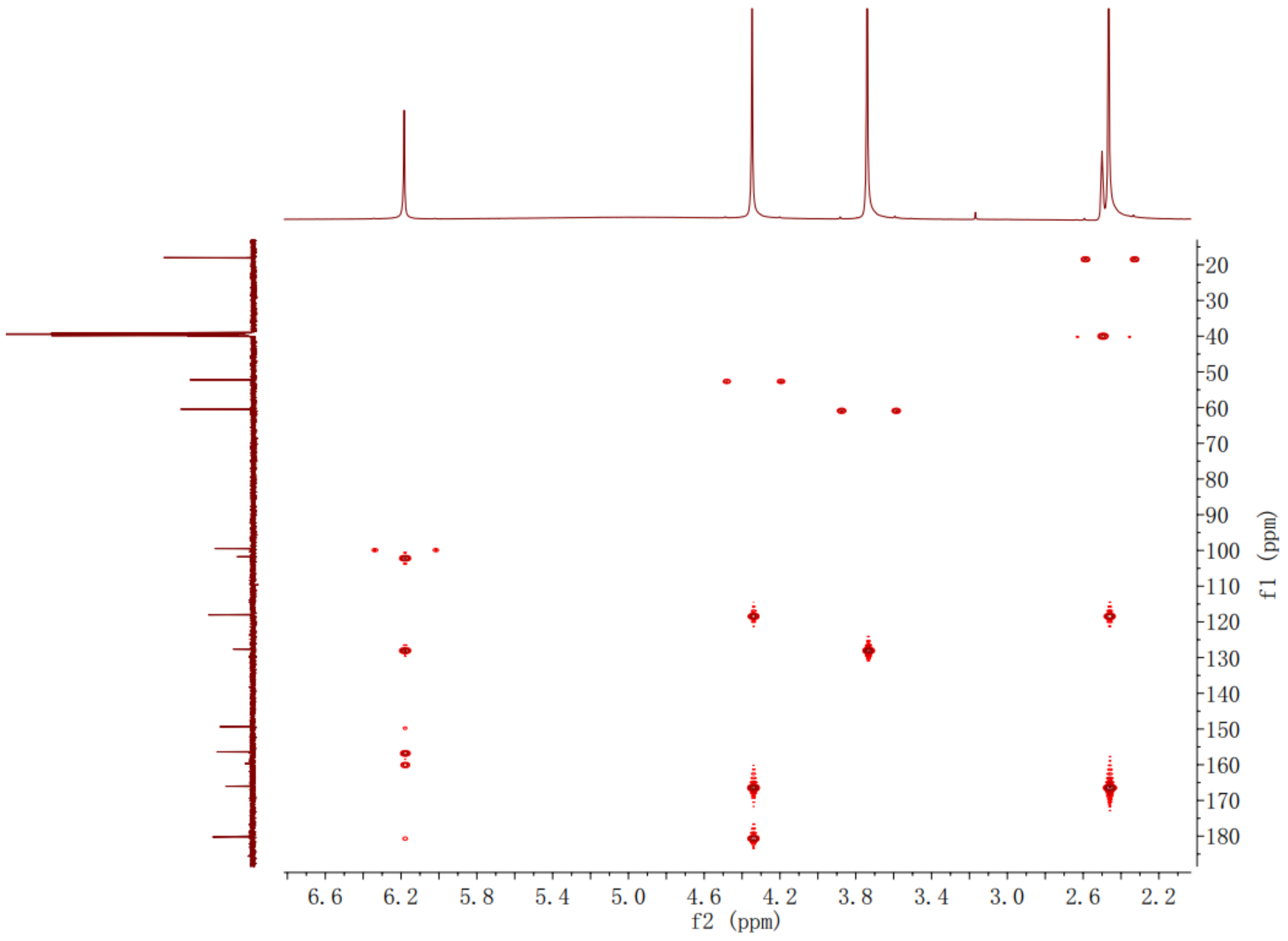

Figure S23. Crystal packing of compound **4** at 296(2) K.

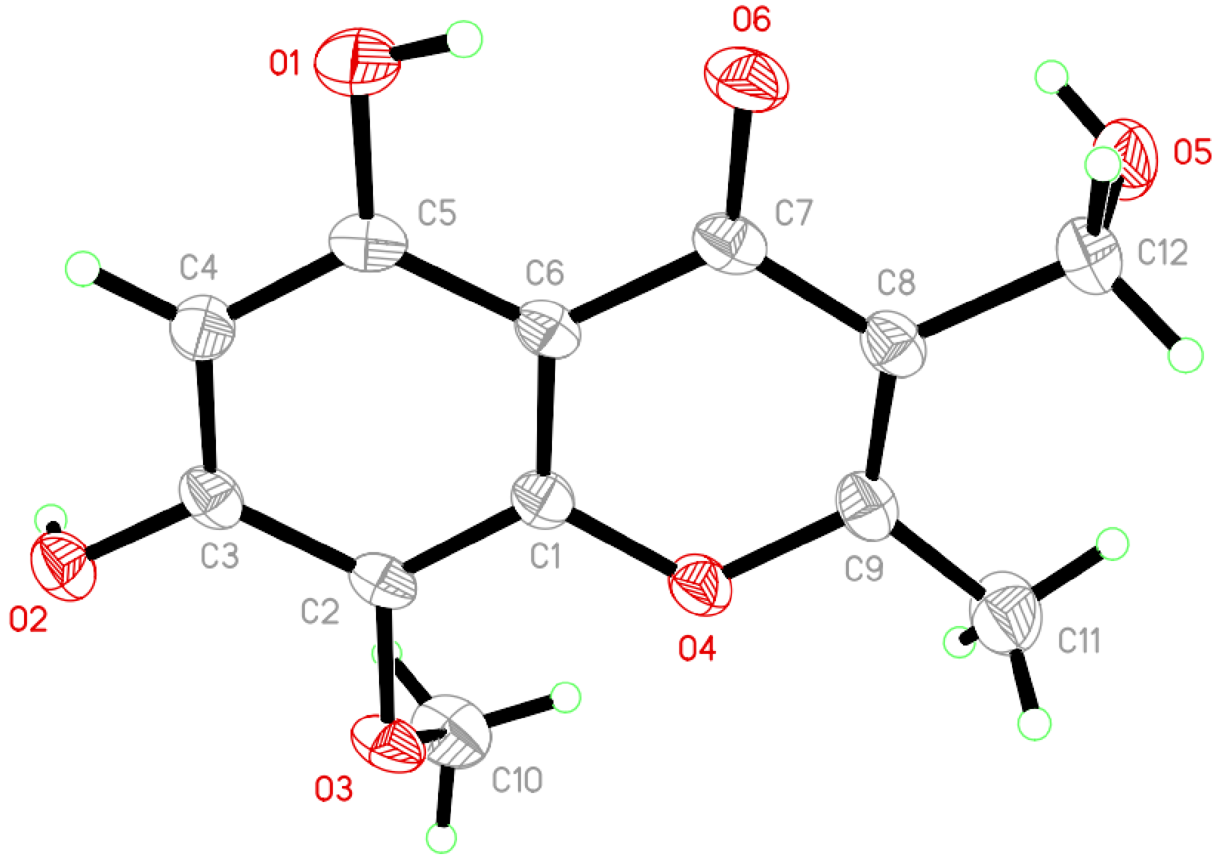

Figure S24. HRESIMS spectrum of compound **5**.

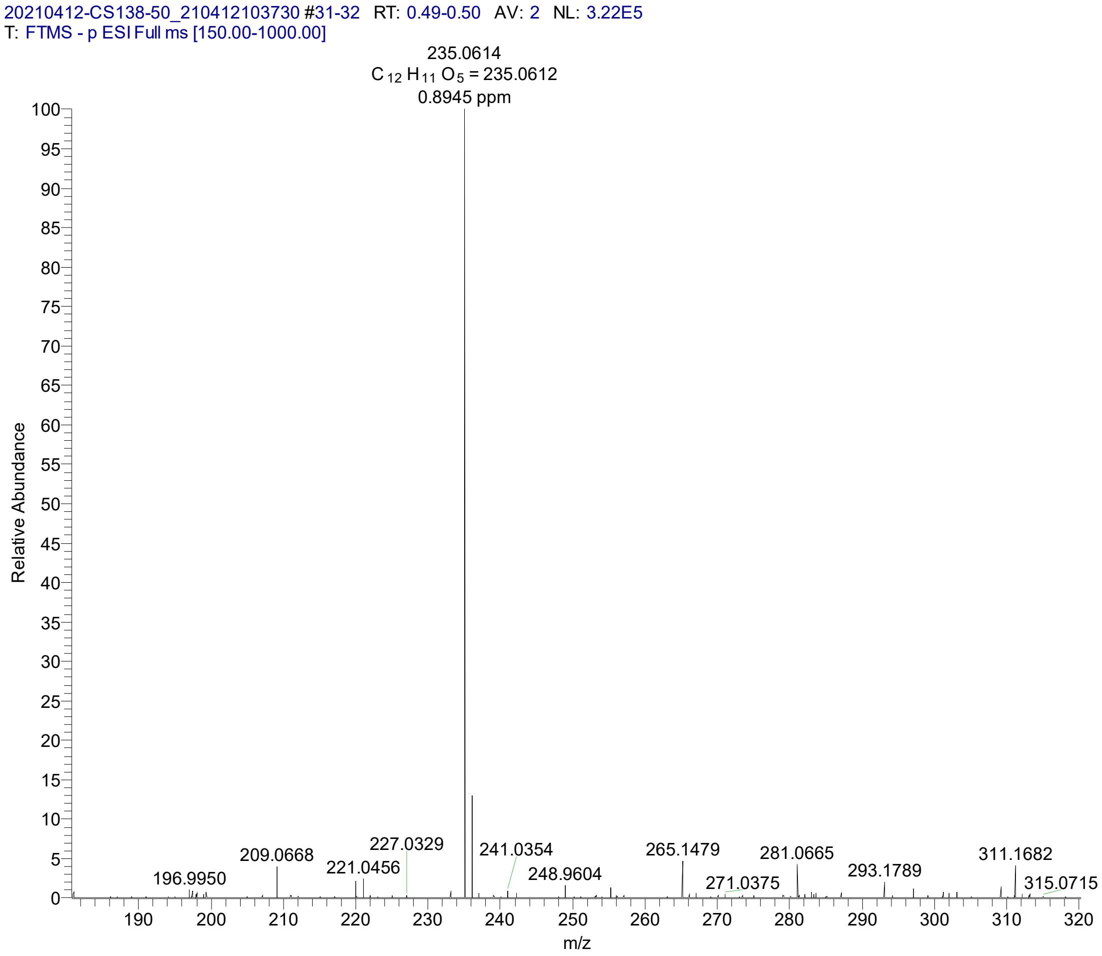

Figure S25. ^{1}H NMR (500 MHz, DMSO-d_6) spectrum of compound **5**.

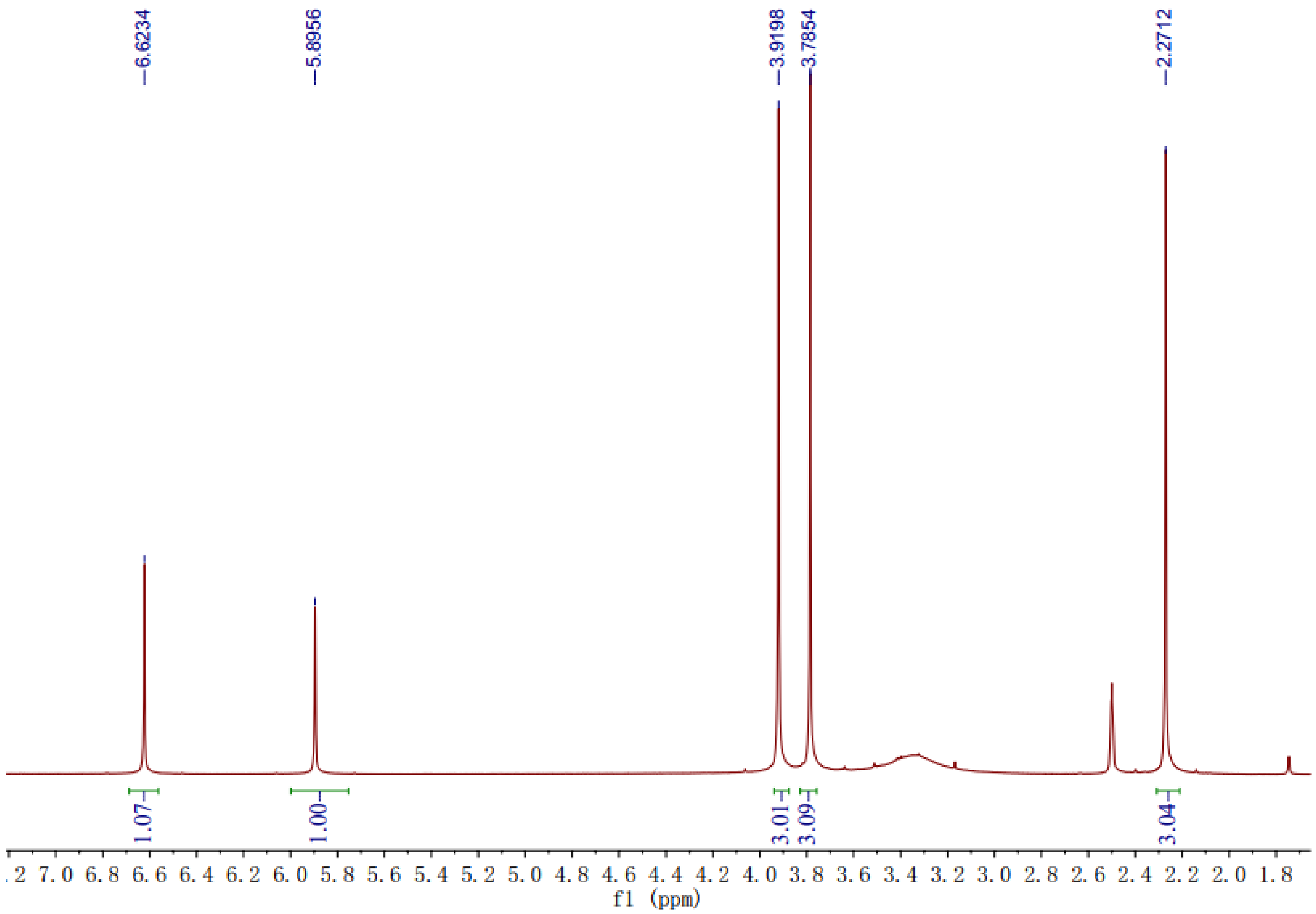

Figure S26. ^{13}C NMR (125 MHz, DMSO-d_6) and DEPT spectra of compound **5**.

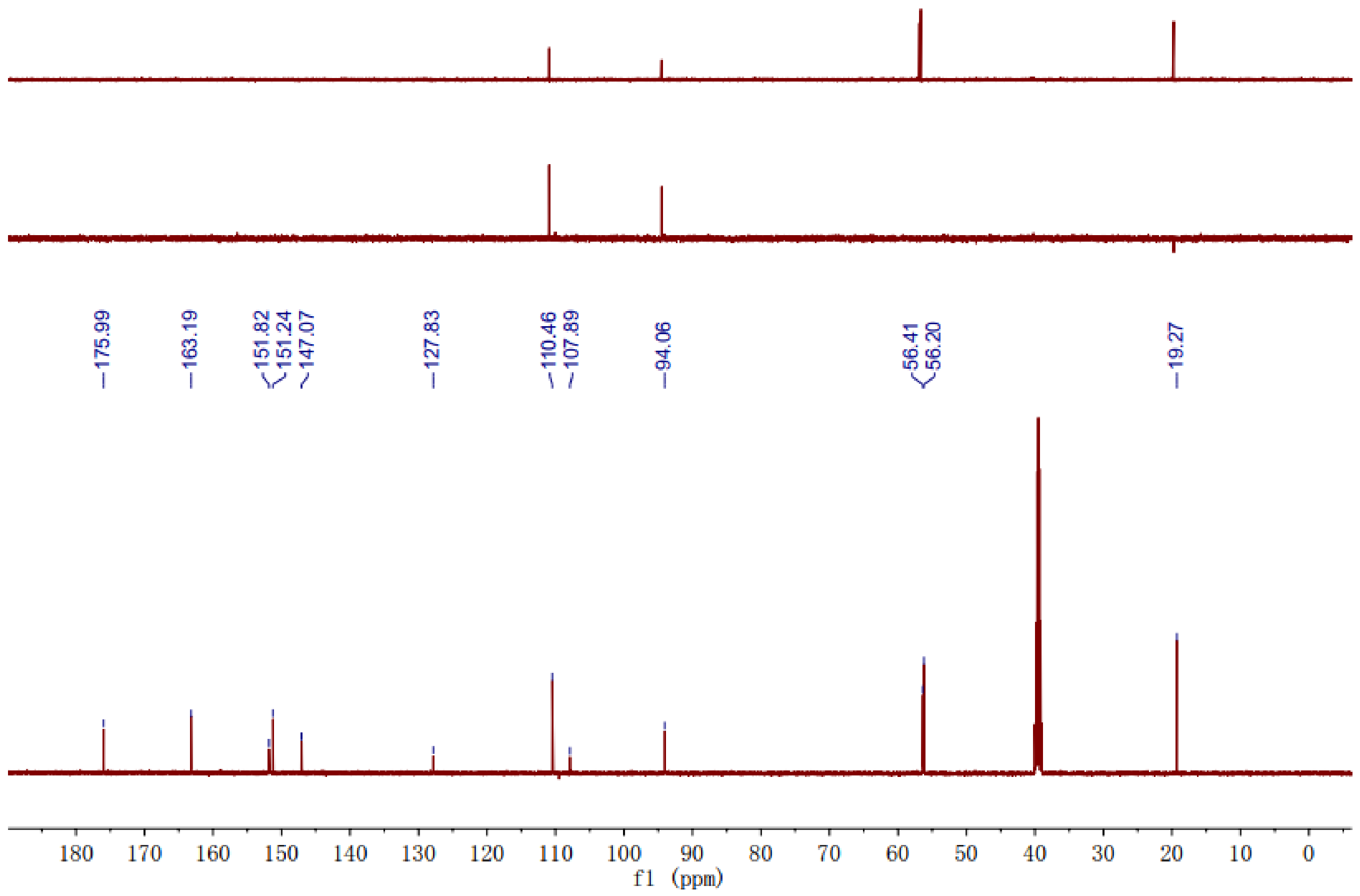

Figure S27. HSQC spectrum of compound **5**.

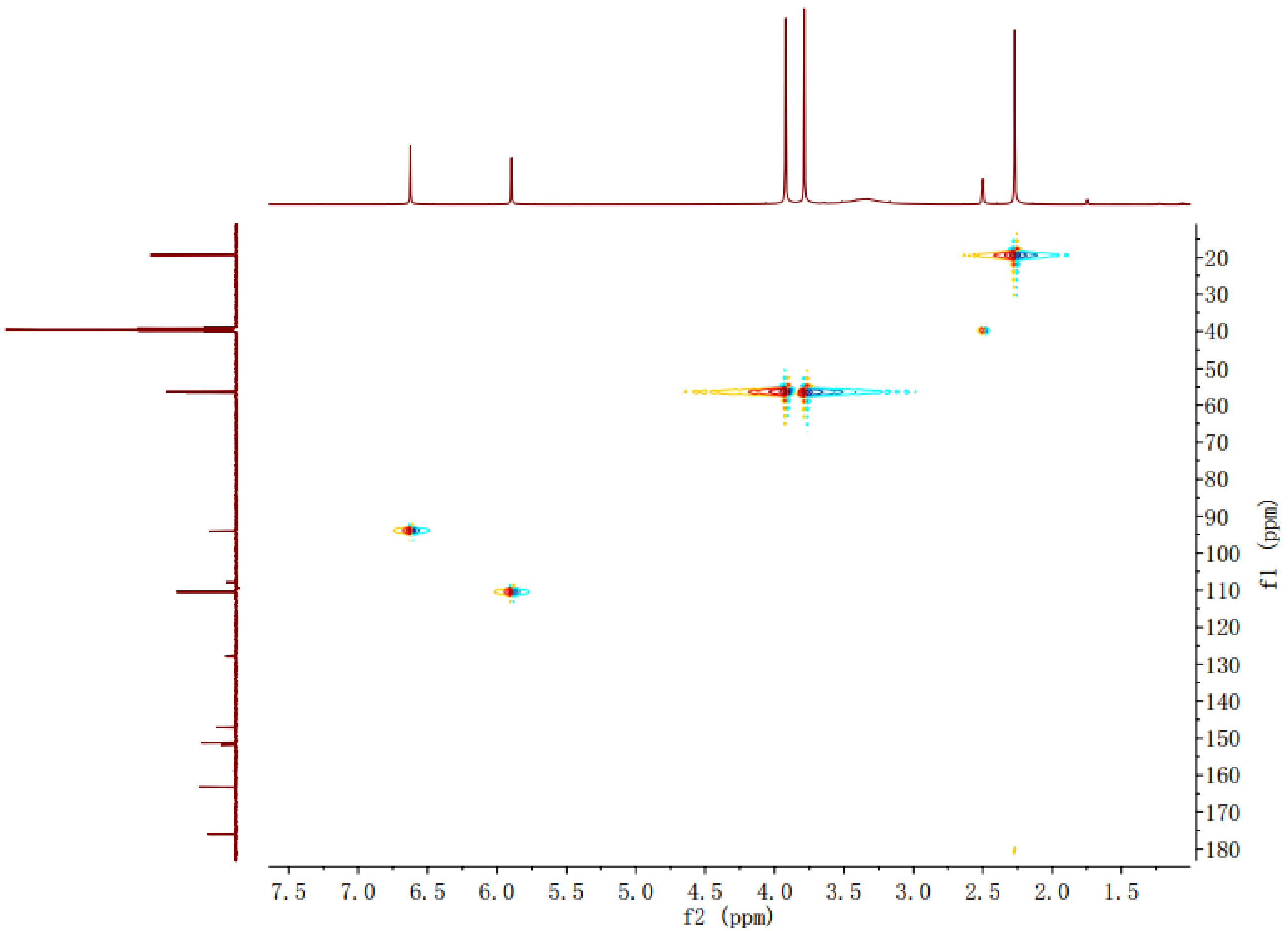

Figure S28. HMBC spectrum of compound **5**.

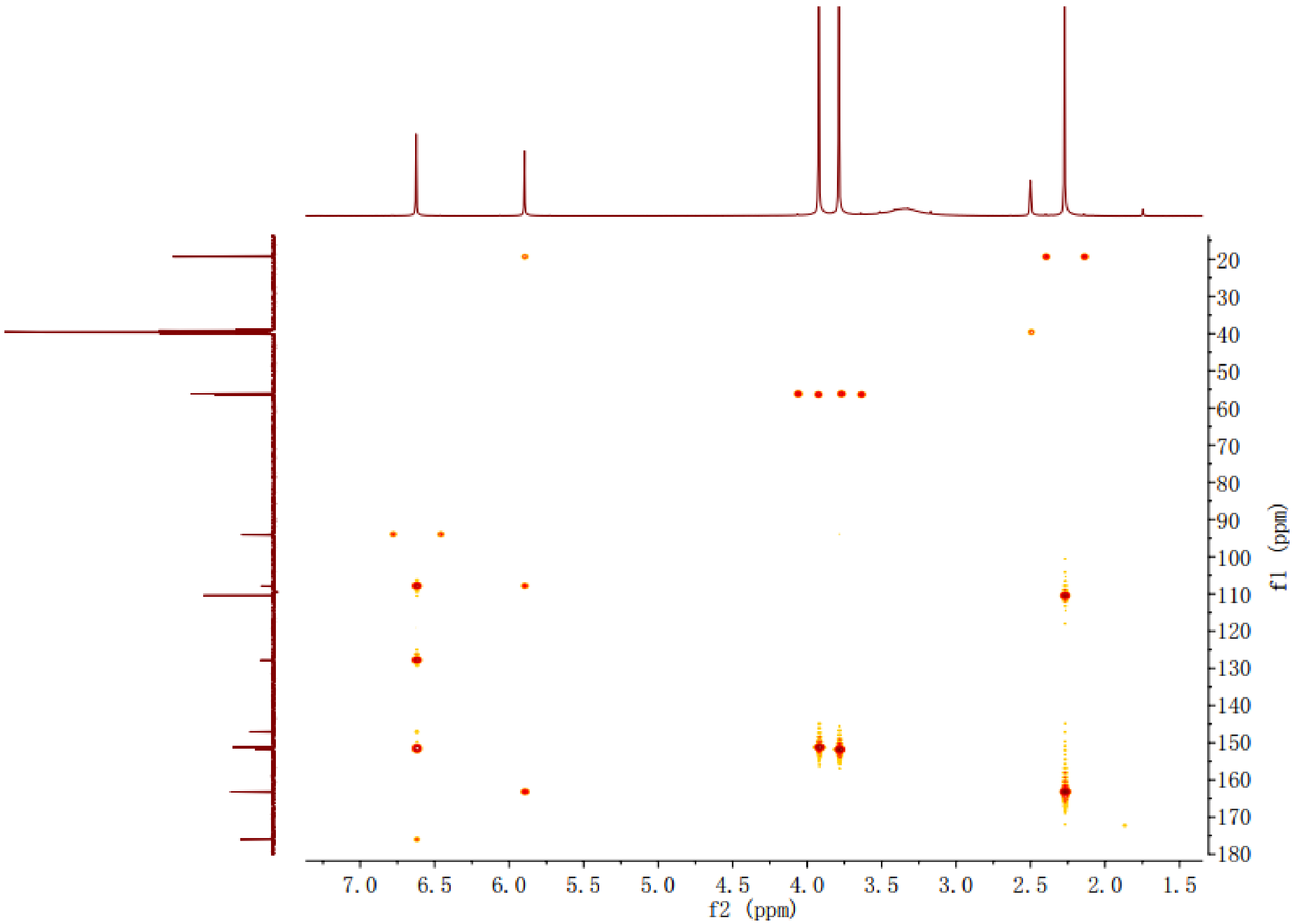

Figure S29. Crystal packing of compound **5** at 298(2) K.

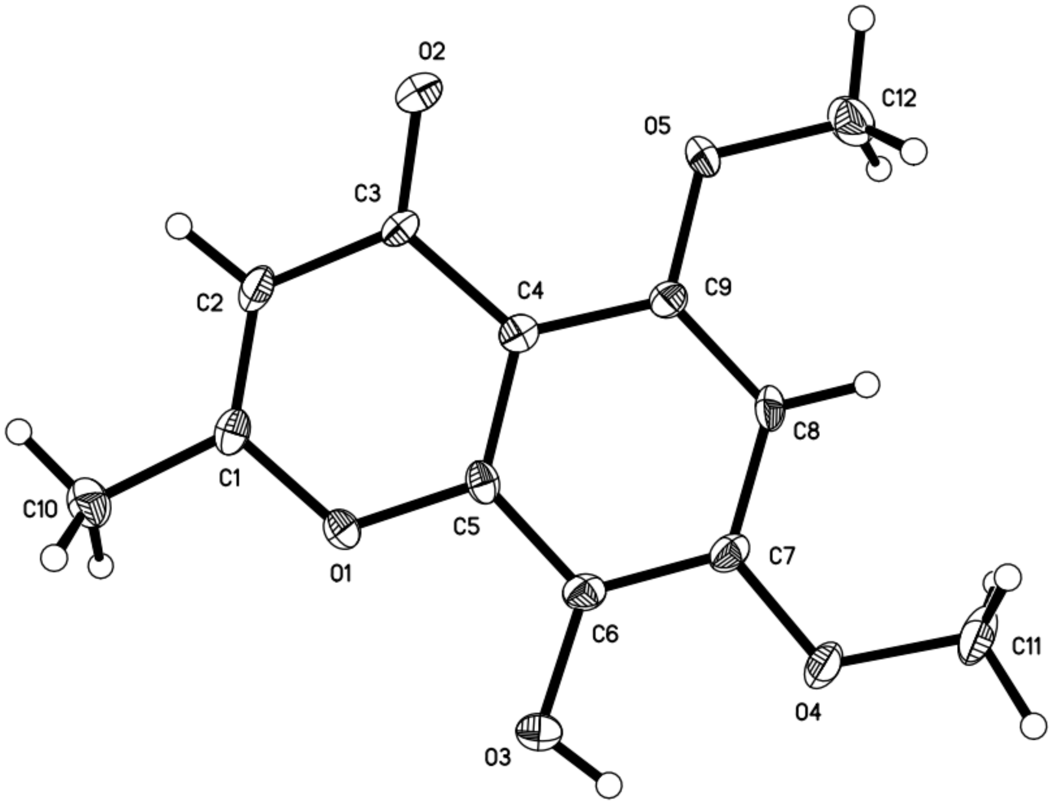

Figure S30. HRESIMS spectrum of compound **6**.

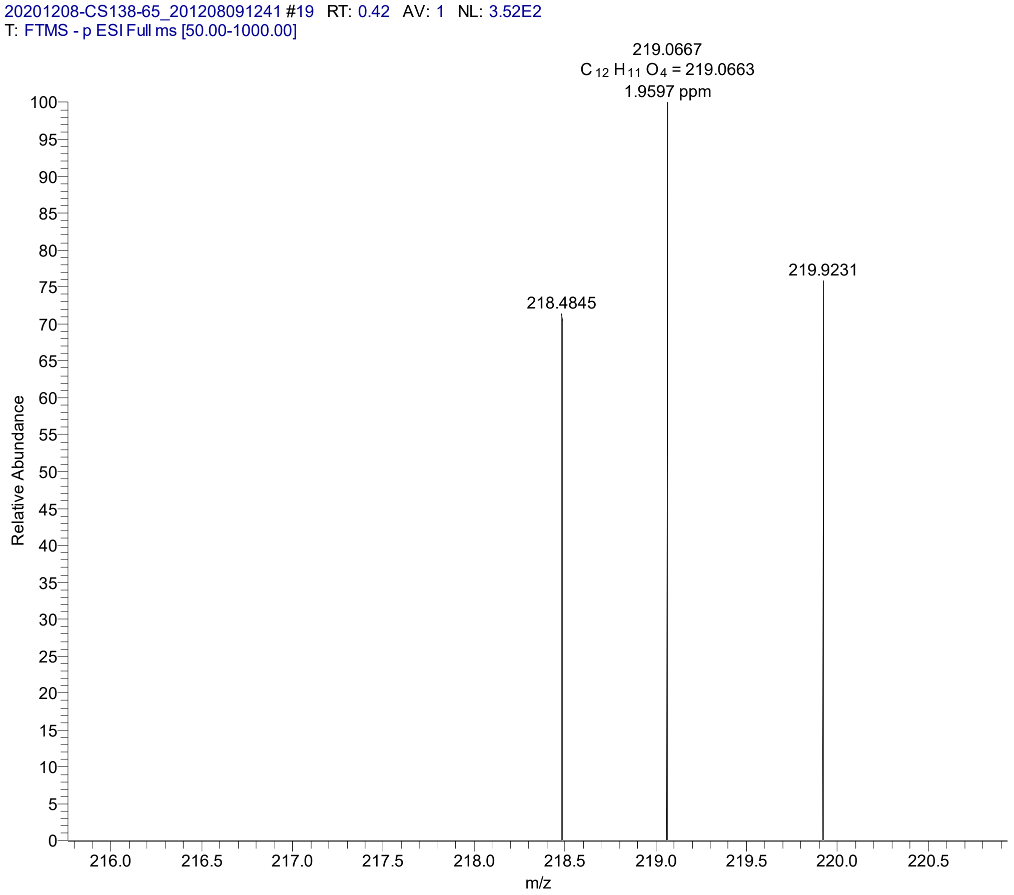

Figure S31. ^{1}H NMR (500 MHz, DMSO-d_6) spectrum of compound **6**.

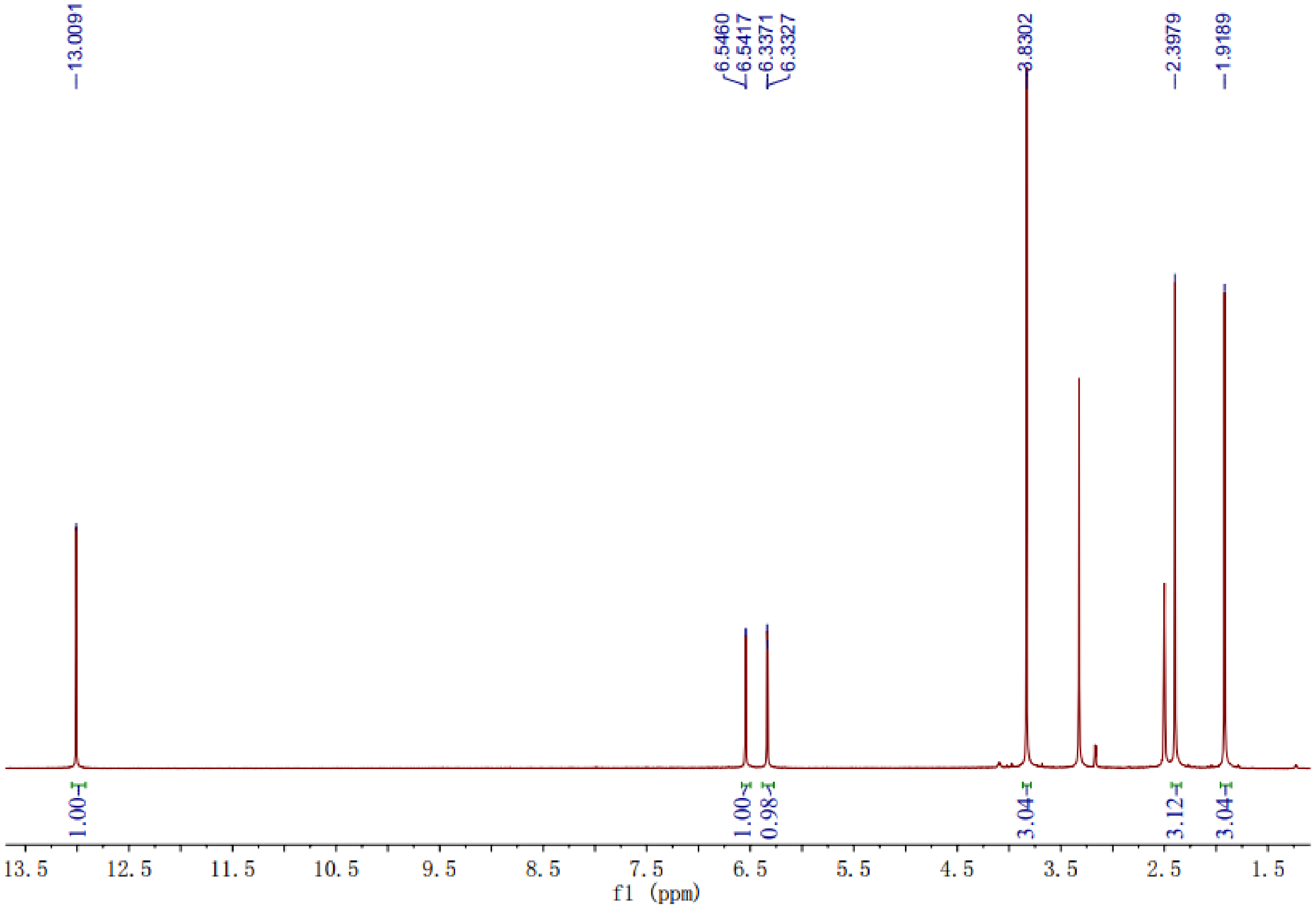

Figure S32. ^{13}C NMR (125 MHz, DMSO-d_6) and DEPT spectra of compound **6**.

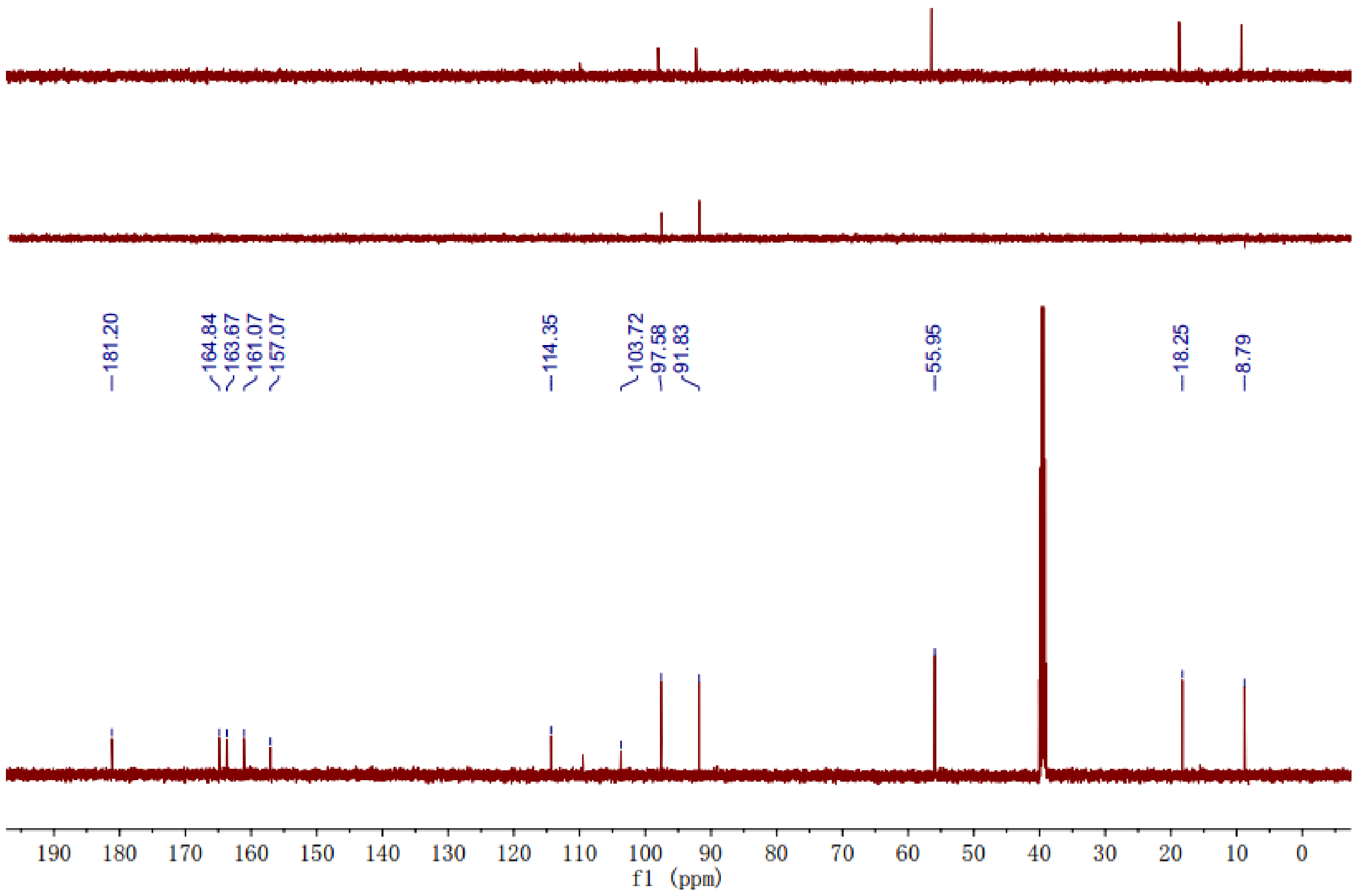

Supplement: Supplementary file 1 [file marinedrugs-20-00529-s001.zip › marinedrugs-1858793-supplementary.pdf]
